# Pan-carcinoma sialyl-Tn-targeting expands CAR therapy to solid tumors

**DOI:** 10.1016/j.xcrm.2025.102350

**Published:** 2025-09-08

**Authors:** Rafaela Abrantes, Christopher Forcados, David J. Warren, Liliana Santos-Ferreira, Karianne Giller Fleten, Emanuel Senra, Ana Filipa Costa, Klara Krpina, Rui Henrique, Ann Magritt Liberg, Puneet Rawat, Pascal Gelebart, Emmet McCormack, Line Bjørge, Ben Davidson, Victor Greiff, Daniela Elena Costea, Filipe Pinto, Kjersti Flatmark, Catarina Gomes, Else Marit Inderberg, Celso A. Reis, Sébastien Wälchli

**Affiliations:** 1 i3S – Instituto de Investigação e Inovação em Saúde, Universidade do Porto, Rua Alfredo Allen 208, 4200-135 Porto, Portugal; 2 IPATIMUP – Instituto de Patologia e Imunologia Molecular da Universidade do Porto, Rua Júlio Amaral de Carvalho 45, 4200-135 Porto, Portugal; 3 ICBAS – Instituto de Ciências Biomédicas Abel Salazar, Universidade do Porto, Rua de Jorge Viterbo Ferreira 228, 4050-313 Porto, Portugal; 4 Translational Research Unit, Department of Cellular Therapy, Oslo University Hospital, Sognsvannsveien 20, 0372 Oslo, Norway; 5 Faculty of Medicine, University of Oslo, Sognsvannsveien 9, 0372 Oslo, Norway; 6 Department of Medical Biochemistry, Oslo University Hospital, Sognsvannsveien 20, 0372 Oslo, Norway; 7 Department of Tumor Biology, Institute for Cancer Research, Norwegian Radium Hospital, Oslo University Hospital, Ullernchausséen 70, 0379 Oslo, Norway; 8 Department of Biosciences, Faculty of Mathematics and Natural Sciences, University of Oslo, Oslo, Norway; 9 Department of Pathology & Cancer Biology and Epigenetics Group – Research Center (CI-IPOP)/RISE@CI-IPOP (Health Research Network), Portuguese Oncology Institute of Porto (IPO Porto)/Porto Comprehensive Cancer Center Raquel Seruca (P.CCC), Rua Dr. António Bernardino de Almeida, 4200-072 Porto, Portugal; 10 Department of Immunology, University of Oslo and Oslo University Hospital, Sognsvannsveien 20, 0372 Oslo, Norway; 11 Department of Clinical Science, Precision Oncology Research Group, University of Bergen, Jonas Lies vei 91, 5009 Bergen, Norway; 12 CCBIO – Centre for Cancer Biomarkers, University of Bergen, Jonas Lies vei 91, 5009 Bergen, Norway; 13 Department of Hematology, Haukeland University Hospital, Jonas Lies vei 65, 5021 Bergen, Norway; 14 Centre for Pharmacy, Department of Clinical Science, University of Bergen, Jonas Lies vei 91, 5009 Bergen, Norway; 15 Department of Obstetrics and Gynecology, Haukeland University Hospital, Jonas Lies vei 65, 5021 Bergen, Norway; 16 Institute for Clinical Medicine, Faculty of Medicine, University of Oslo, Sognsvannsveien 9, 0372 Oslo, Norway; 17 Department of Pathology, Division of Laboratory Medicine, Oslo University Hospital, Sognsvannsveien 20, 0372 Oslo, Norway; 18 Department of Clinical Medicine, University of Bergen, Jonas Lies vei 91, 5009 Bergen, Norway; 19 Gade Laboratory for Pathology, Haukeland University Hospital, Jonas Lies vei 65, 5021 Bergen, Norway; 20 Department of Surgical Oncology, Norwegian Radium Hospital, Oslo University Hospital, Ullernchausséen 70, 0379 Oslo, Norway; 21 Faculty of Medicine, Institute of Clinical Medicine, University of Oslo, Sognsvannsveien 9, 0372 Oslo, Norway; 22 FMUP – Faculdade de Medicina da Universidade do Porto, Alameda Prof. Hernâni Monteiro, 4200-319 Porto, Portugal

**Keywords:** immunotherapy, CAR T cells, glycosylation, sialyl-Tn, cancer, antibodies, glycobiology, solid tumors

## Abstract

Accurate identification of tumor-specific markers is vital for developing chimeric antigen receptor (CAR)-based therapies. While cell surface antigens are seldom cancer-restricted, their post-translational modifications (PTMs), particularly aberrant carbohydrate structures, offer attractive alternatives. Among these, the sialyl-Tn (STn) antigen stands out for its prevalent presence in various epithelial tumors. Although monoclonal antibodies (mAbs) against STn have been developed, their clinical application has been hindered by concerns regarding specificity. Herein, we describe AM52.1, a mAb with unprecedented specificity for STn and lack of reactivity with healthy tissues. The single-chain variable fragment (scFv) of AM52.1 was assembled into a second-generation CAR scaffold. AM52.1CAR T cells efficiently targeted STn-expressing cancer cell lines and patient-derived organoids (PDOs), while sparing STn-negative cells. In further preclinical models, AM52.1CAR T cells robustly controlled gastric and tubo-ovarian tumors, as well as colorectal cancer mucinous peritoneal metastases, highlighting their strong therapeutic potential for targeting and managing complex solid tumors.

## Highlights

• AM52.1 monoclonal antibody binds exclusively to sialyl-Tn (STn)
• AM52.1CAR T cells eradicate STn-positive tumor cells, even at low antigen density
• AM52.1CAR T cells control tumor growth *in vitro* and *in vivo*
• AM52.1CAR T cells control peritoneal metastases in a complex PDX model

## Graphical abstract

**Figure:**
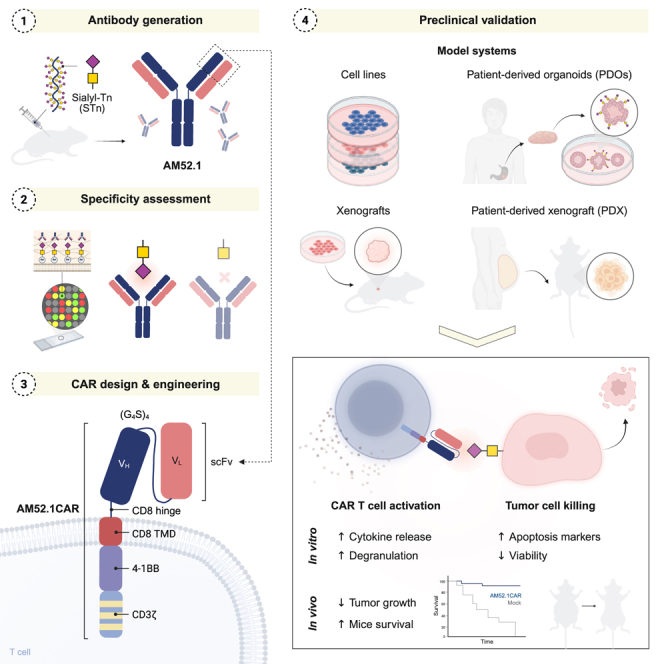

## Teaser

Anti-glycan antibodies often show some degree of cross-reactivity. Abrantes et al. report AM52.1, an antibody with exceptional specificity for the tumor-associated sialyl-Tn (STn) antigen. In different solid tumor models, AM52.1CAR T cells effectively control STn-expressing gastric and tubo-ovarian cancers, as well as colorectal peritoneal metastases, without evidence of off-tumor toxicity.

## Introduction

Chimeric antigen receptor (CAR) T cell therapy has garnered significant attention for its remarkable clinical success in the treatment of hematological malignancies.^1^^,^^2^^,^^3^ However, the successful translation of CAR T cell immunotherapy to treat solid tumors remains a difficult task.^4^^,^^5^^,^^6^^,^^7^ Aside from the challenging accessibility of certain anatomical regions and the presence of a highly immunosuppressive tumor microenvironment (TME), carcinomas usually lack specific molecular targets, as most cancer cell markers are also expressed in healthy epithelial cells.^8^ Therefore, it is imperative to specifically target surface antigens whose expression is restricted to cancer cells to generate an effective CAR-based therapy.

Glycans are complex carbohydrate structures attached to a variety of molecular carriers frequently expressed on the surface of mammalian cells, and play pivotal roles in many cellular processes, including cell adhesion, signaling, and immune recognition.^9^^,^^10^ They have emerged as compelling targets for cancer therapeutics, owing to their aberrant yet specific expression patterns on cancer cells.^11^^,^^12^^,^^13^ Indeed, the well-established and highly specific expression of several cancer-associated glycan epitopes on the surface of cancer cells makes them appealing molecular targets for novel CAR T formulations. One such glycan structure is the Thomsen-Friedenreich-related sialyl-Tn (STn) antigen (NeuAcα2,6-GalNAcα1-*O*-Ser/Thr),^14^ a prematurely truncated *O*-glycan structure, which has gained attention for its prominent and highly specific expression profile in various epithelial tumors, including gastric,^15^ colorectal,^15^^,^^16^ tubo-ovarian,^17^ prostate,^18^ and pancreatic^19^ cancer, while its detection is rare or absent in normal tissues. Consequently, targeting STn with CAR T cells holds tremendous potential as an effective treatment against a wide range of solid tumors.

The specificity of a CAR molecule primarily lies within its antigen-binding domain, typically composed of a single chain variable fragment (scFv) derived from an antibody.^20^ While several murine monoclonal antibodies (mAbs) have been engineered to target STn,^21^^,^^22^^,^^23^^,^^24^ they have been mainly applied in tissue-based diagnostics. Indeed, these mAbs were shown to have some degree of binding to non-STn glycan motifs and to be dependent on the surrounding peptidic context.^25^^,^^26^ This lack of absolute specificity poses challenges to the development of STn-directed CAR T cell therapies, as off-target effects could potentially compromise therapeutic efficacy and patient’s safety. For instance, while the CC49 mAb has been studied in both preclinical and clinical settings, its translation into a first-generation CAR T cell therapy has faced significant challenges, as evidenced by limited anti-tumor responses in a clinical trial in colorectal cancer (CRC).^27^ More recently, a second-generation CC49-based CAR has demonstrated improved preclinical efficacy and is currently under clinical evaluation for chemoresistant advanced ovarian cancer (OC) (NCT05225363).^28^ Similarly, a modular CAR targeting both STn and α2,6-sialylated core 1 structures has shown effectiveness in bladder and breast cancer models.^29^^,^^30^

Herein, we describe the generation and characterization of two STn-targeting mAbs using hybridoma technology—AM51.1 and AM52.1. We further present CAR T cells based on AM52.1 mAb, AM52.1CAR T cells, which efficiently identify and kill STn-expressing cancer cells both *in vitro* and in mouse xenograft models, while displaying negligible reactivity toward STn-negative cells. Additionally, AM52.1CAR T cells demonstrated strong efficacy in challenging preclinical models, including patient-derived organoids (PDOs) and xenografts (PDXs).

## Results

### AM52.1 is a pan-carcinoma antibody with specific recognition of sialyl-Tn

**Figure 1:**
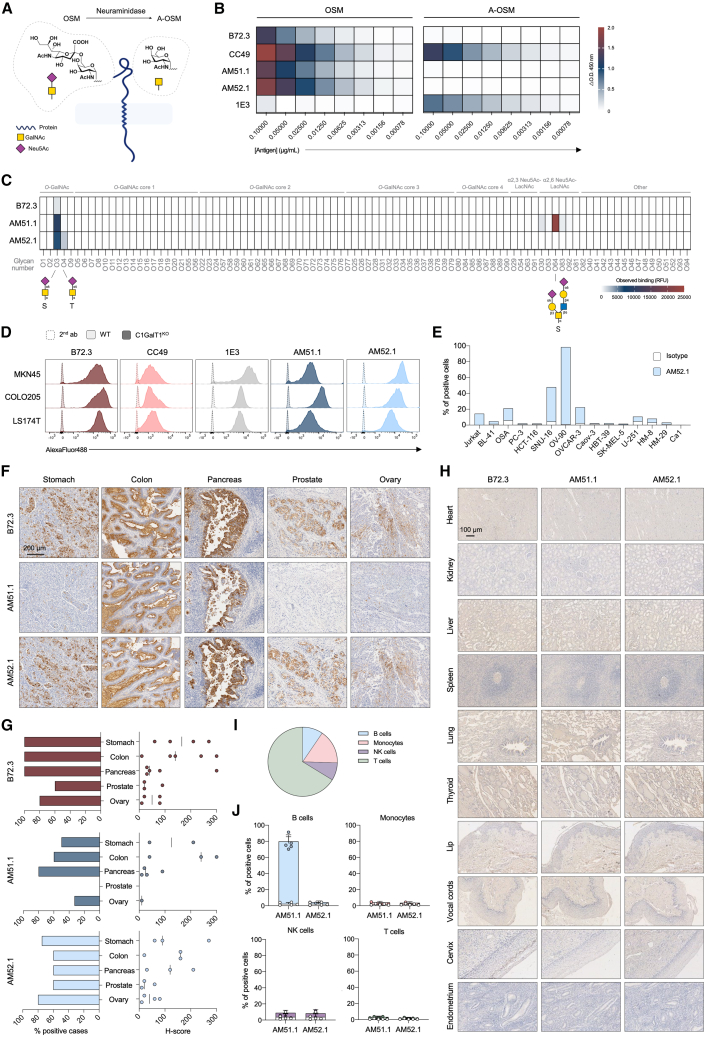
AM52.1 antibody shows specific binding to sialyl-Tn (A) Schematic illustration of the predominant glycan structures in ovine submaxillary mucin (OSM) and its desialylated form, Asialo-OSM (A-OSM). (B) Binding of AM mAbs to varying concentrations of OSM and A-OSM, measured by indirect enzyme-linked immunosorbent assay (ELISA). B72.3, CC49, and 1E3 antibodies were included as controls. (C) Binding specificity of AM mAbs to various sialyl-Tn (STn)-related *O*-glycan structures, as measured by glycan microarray analysis; 10 μg/mL of each antibody were used. B72.3 mAb served as a control for the assay. Refer to Figure S1A for a comprehensive list of the analyzed glycan structures. (D) Gastrointestinal cancer wild-type (WT) and core 1 β1,3-galactosyltransferase (C1GalT1) knockout (KO) cells were stained for flow cytometry using the AM mAbs, and well-established anti-STn and anti-Tn antibodies as controls. Histograms represent the cell populations stained with the annotated antibodies. (E) Cell lines of different origins (lymphoma, osteosarcoma, melanoma, glioblastoma, prostate, colorectal, gastric, ovarian, breast, and oral cancer) were stained with AM52.1. Representative flow cytometry plots of isotype control and AM52.1 staining are shown. (F) AM51.1 and AM52.1 antibodies, alongside with the positive control B72.3, were used to stain tissue from gastric, colon, pancreatic, prostate, and ovarian carcinomas. Sections show representative staining of tumor tissue with the annotated antibodies. Scale bar corresponds to 200 μm. (G) Graphs depicting the percentage of carcinoma sections exhibiting positive staining for each antibody tested and the corresponding H-score for each tissue type. (H) Healthy tissues (heart, kidney, liver, spleen, lung, thyroid, lip, vocal cords, cervix, and endometrium) were stained with the AM antibodies and the B72.3 positive control. Scale bar corresponds to 100 μm. (I) Pie chart representing cell population distribution in human peripheral blood mononuclear cells (PBMCs) from healthy donors (*n* = 5) stained with the AM mAbs. (J) Histograms depicting the percentage distribution of various immune cell populations stained with AM51.1 and AM52.1. Data are represented as mean ± standard deviation (SD).

STn-directed mAbs were generated by using ovine submaxillary mucin (OSM), a highly glycosylated protein containing 98% of STn as glycan content, as the immunogen (Figure 1A). An initial enzyme-linked immunosorbent assay (ELISA) screening of 1,000 hybridoma clones identified AM51.1 and AM52.1 as those exhibiting the highest specific binding (data not shown). Comparative analysis of these clones with established anti-STn and anti-Tn mAbs, B72.3 and CC49, and 1E3, respectively, confirmed that AM51.1 and AM52.1 bind specifically and in a dose-dependent manner to STn-glycosylated OSM, without cross-reacting with Tn (Figure 1B). To determine the exact binding epitope of each antibody, the AM mAbs were probed against an *O*-glycan array containing 94 distinct chemically synthesized and well-characterized *O*-glycan structures related to STn (Figure S1A). AM52.1 showed a remarkable specificity for STn independently of the glycosylated amino acid, even at lower concentrations (Figures 1C and S1B). In contrast, AM51.1 also recognized α2,6-linked sialic acids in the context of poly-*N*-acetyllactosamine sequences. As a control, the B72.3 antibody was tested in parallel, and its specificity for STn within serine residues was confirmed.

The AM mAbs’ ability to detect STn in the context of different cancer cell line models was evaluated. Simulation of truncated *O*-glycans expression was achieved by knocking out the core 1 β1,3-galactosyltransferase (C1GalT1) using clustered regularly interspaced short palindromic repeats (CRISPR)-Cas9.^31^ Both AM antibodies exhibited selective binding to C1GalT1-deficient cells, while showing no reactivity toward the parental cells (Figure 1D). These findings were supported by western blot analysis and immunofluorescent labeling (Figures S2A and S2B). Additionally, to confirm the dependence of AM mAb binding on the presence of sialic acids, we used neuraminidase-treated COLO205 protein lysate blots in western blotting, which revealed a drastic loss of signal under treated conditions (Figure S2C). We then confirmed endogenous STn expression in several cell lines, including the gastric cancer (GC) cell line SNU-16 and the OC cell lines OV-90 and OVCAR-3 (Figure 1E). These cells were also stained after neuraminidase digestion with the different anti-STn mAbs (Figures S2D–S2F), with AM52.1 showing to be more sensitive to the absence of sialic acids than B72.3 or CC49, indicating increased specificity toward the sialylated GalNAc structure. In addition, AM51.1 mAb showed minimal reactivity toward the naturally STn-expressing cell lines tested. These observations were further corroborated by western blotting and immunostaining analyses (Figures S2G and S2H), where AM51.1 displayed minimal STn detection.

We next examined AM mAbs’ selectivity across a panel of human epithelial tissues in normal and tumor settings. The staining patterns observed for the tested mAbs were consistent concerning the cell type and subcellular location within the same carcinoma tissue specimens (Figure 1F). Importantly, despite heterogeneous tissue reactivity having been observed among distinct tumor types (Figure 1G; Table S1), the staining patterns of B72.3 and AM52.1 were notably overlapping. Nonetheless, AM52.1 depicted immunoreactivity in a large proportion of carcinomas, including gastric (80%), colon (60%), pancreatic (60%), prostate (60%), and tubo-ovarian (80%) cancers. AM51.1, although displaying weaker detection capability, also stained gastric (50%), colon (60%), pancreatic (80%), and tubo-ovarian (33%) cancer tissues. Importantly, AM52.1 did not react with any healthy epithelial tissues, immune tissue (spleen), or peripheral blood mononuclear cells (PBMCs) (Figures 1H–1J), whereas AM51.1 cross-reacted with a large part of the B cell population (Figures 1I and 1J).

Taken together, these data demonstrate that AM52.1 exhibits higher specificity for the cancer-associated STn antigen compared to the currently available anti-STn antibodies, while AM51.1, despite being isolated from the same screening, displays cross-reactivity with other sialylated structures and appears to rely on a specific STn presentation context.

### *In silico* analysis reveals sialyl-Tn-restricted binding of AM52.1-derived single-chain variable fragment

**Figure 2:**
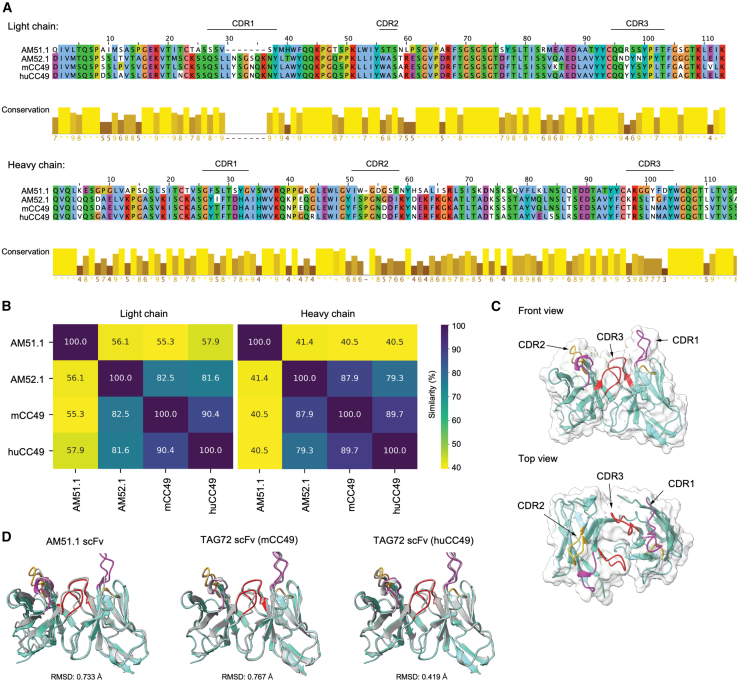
Sequence and structural analysis indicating a sialyl-Tn-restricted binding of the AM52.1-based single-chain variable fragment (A) Multiple sequence alignment (MSA) for the single-chain variable fragment (scFv) region derived from various anti-sialyl-Tn (STn) monoclonal antibodies (mAbs). The aligned light and heavy chain sequences were visualized using the Clustal Color Scheme in Jalview, grouping residues based on their similarity. The complementarity-determining regions (CDR) regions were mapped on the alignment and annotated using the ImMunoGeneTics (IMGT) database numbering scheme. (B) Percentage of sequence identity calculated for the light and heavy chains of the scFv derived from the investigated antibodies, using MSA. (C) Front and top view of the AM52.1-based scFv structure with the CDR regions mapped (CDR1: violet; CDR2: yellow; CDR3: red). The antibody surface is illustrated using a white-colored volume mesh. (D) The AM52.1-based scFv (cyan color) was structurally aligned with the scFv derived from the other anti-STn mAbs (gray color). Root-mean-square deviation (RMSD) values are given below the structure for each pair.

To further evaluate the therapeutic promise held by the AM mAbs, we engineered AM mAbs-based scFvs for subsequent CAR generation, and conducted a comparative analysis of their sequences and structures with those derived from the well-studied native CC49 clone (murine CC49, mCC49) and its humanized version (huCC49).^32^ The sequences showed very high similarity to the germline sequences identified by the ImMunoGeneTics (IMGT) database. The multiple sequence alignment (MSA) revealed significant differences in both the light and heavy chains from the complementarity-determining region 3 (CDR3) (Figure 2A). However, the AM52.1-derived scFv exhibited greater similarity to the mCC49 (sequence identity ≥80% for both chains) (Figure 2B). Regarding the AM51.1-derived scFv, while the light chain had relatively moderate sequence similarity (≥50%), it has demonstrated low sequence similarity with other scFvs in the heavy chains (≤43%) (Figure 2B). Further investigating into the impact of sequence variations on structural resemblance, we used ABodyBuilder2^33^ and IgFold^34^ to model the scFv structures built from each antibody. Notably, the predicted scFv structures from both methods exhibited excellent agreement (root-mean-square deviation [RMSD] < 0.5 Å) (Figure S3). Subsequently, structures generated by ABodyBuilder2 were further used as representative models for each scFv due to their minimal prediction error (average 0.23 Å across the framework regions and CDRs of all scFvs) (Table S2). Unexpectedly, comparative analysis unveiled a notably greater structural resemblance between the AM52.1-derived scFv (Figure 2C) and the huCC49, in contrast to the mCC49-derived scFv (Figure 2D). In summary, our *in silico* analysis shows high sequence and structural similarity between the scFv derived from the AM52.1 antibody and the one based on the well-studied CC49, both murine and humanized, reinforcing its potential for targeted immunotherapy against STn-expressing tumors.

### Unlike AM51.1CAR, AM52.1CAR efficiently redirects primary T cells

**Figure 3:**
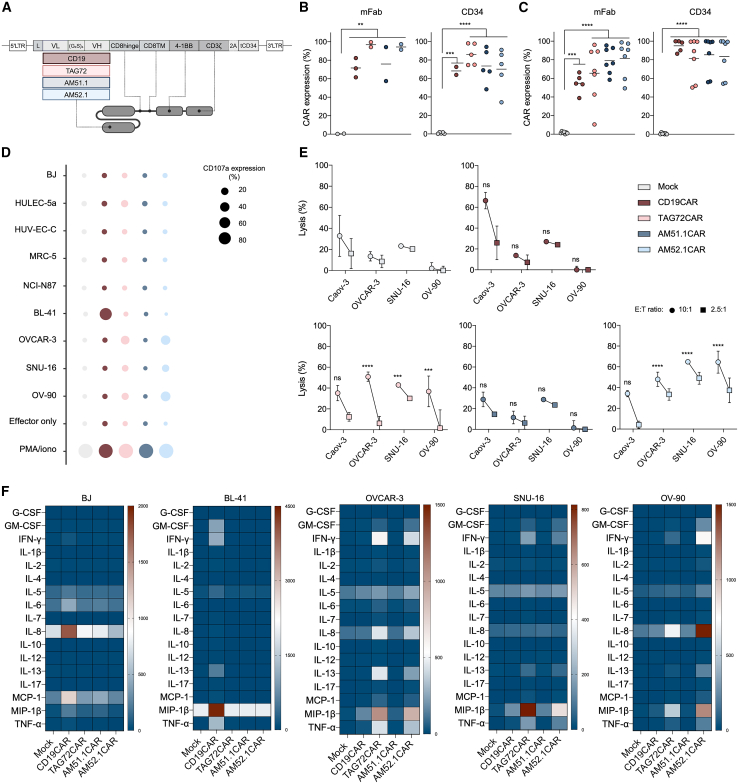
AM52.1-derived CAR T cells reveal specific cytotoxicity against sialyl-Tn-expressing cancer cell lines (A) Illustrative schematic of CAR construct design. (B and C) (B) Jurkat and (C) primary T cells were stably transduced with CAR constructs, and CAR expression was assessed through quantification of murine fragment antigen-binding (mFab) region and CD34 expression. *n* = 2–3 for the Jurkat and *n* = 5–7 for the healthy donors. (D) Degranulation activity of CD4^+^ and CD8^+^ CAR T cells co-cultured with sialyl-Tn (STn)-negative and STn-positive target cells at an effector:target (E:T) ratio of 1:2 for 24 h. *n* = 3 healthy donors. (E) CAR T cell-specific cytotoxicity assessed by a bioluminescence-based killing assay. CAR T cells were co-cultured with cell lines expressing various STn levels at the indicated E:T ratios. Data correspond to the 12-h time point. *n* = 3, *n* = 5, and *n* = 2 healthy donors for Caov-3, SNU-16, and OVCAR-3 and OV-90, respectively. (F) Cytokine secretion following 24-h co-culture of CAR T cells with STn-expressing and non-expressing cells. The E:T ratio used was of 1:1. *n* = 3 healthy donors. Data are represented as mean ± standard deviation (SD). Statistical significance was determined using paired t test for (A) and (B), and two-way ANOVA for (E). ∗*p* ≤ 0.05, ∗∗*p* ≤ 0.01, ∗∗∗*p* ≤ 0.001, and ∗∗∗∗*p* ≤ 0.0001.

To evaluate the therapeutic efficacy of the AM antibodies, scFvs were designed and subcloned into a CAR scaffold vector to obtain second-generation constructs (Figure 3A). Monitoring of the transfection efficiency was achieved by linking the CAR coding sequence to a truncated CD34 (tCD34) tag.^35^ For comparison, we cloned the published STn-directed TAG72CAR, derived from the CC49 antibody, and an unrelated CAR targeting CD19 (CD19CAR) into the same vector. CAR expression was validated in both J-76-NFAT-GFP and primary human T cells using anti-Fab and anti-CD34 antibodies (Figures 3B and 3C). All constructs were equally expressed at the membrane without affecting T cell expansion (Figures S4A and S4B), suggesting that the scFvs were stable, properly folded, and non-toxic. Functional evaluation using J-76-NFAT-GFP cells co-cultured with STn-negative and STn-positive cancer cells revealed that both AM-derived CARs were active and specific (Figure S4C). In parallel, degranulation assays with primary T cells demonstrated T cell activation upon antigen recognition, with AM521.CAR T cells upregulating CD107a in response to STn-positive cancer cells, but not to STn-negative cancer or normal cell lines (Figures 3D and S4D). In contrast, AM51.1CAR T cells showed no CD107a expression, while TAG72CAR T cells responded to STn-positive cells, albeit to a lesser extent than AM52.1CAR T cells, and moderately reacted to the STn-negative HULEC-5a microvascular endothelial cell line. Cytotoxicity assays further demonstrated the efficacy of AM52.1CAR T cells against STn-expressing targets, showing superiority over TAG72CAR T cells, particularly at lower effector:target (E:T) ratios, whereas AM51.1CAR T cells showed no reactivity (Figure 3E). Importantly, AM52.1CAR T cells remained inactive toward STn-negative targets (Figures 3E and S4E). We next analyzed cytokine secretion upon CAR engagement using a 17-plex immunoassay. AM52.1CAR T cells produced pro-inflammatory cytokines, including macrophage inflammatory protein 1 beta (MIP-1β), interleukin (IL-8), interferon gamma (IFN-γ), tumor necrosis factor alpha (TNF-α), and IL-13, in response to STn-positive cancer cells (Figure 3F and S4F). Notably, AM52.1CAR T cells were sensitive enough to secrete cytokines in response to cancer cells expressing relatively low STn levels, such as OVCAR-3. Importantly, no cytokines were released upon exposure to STn-negative cancer or normal cell lines. Together, these functional data confirm that the AM52.1 mAb can be successfully converted into an efficient CAR, retaining its specificity against STn.

**Figure 4:**
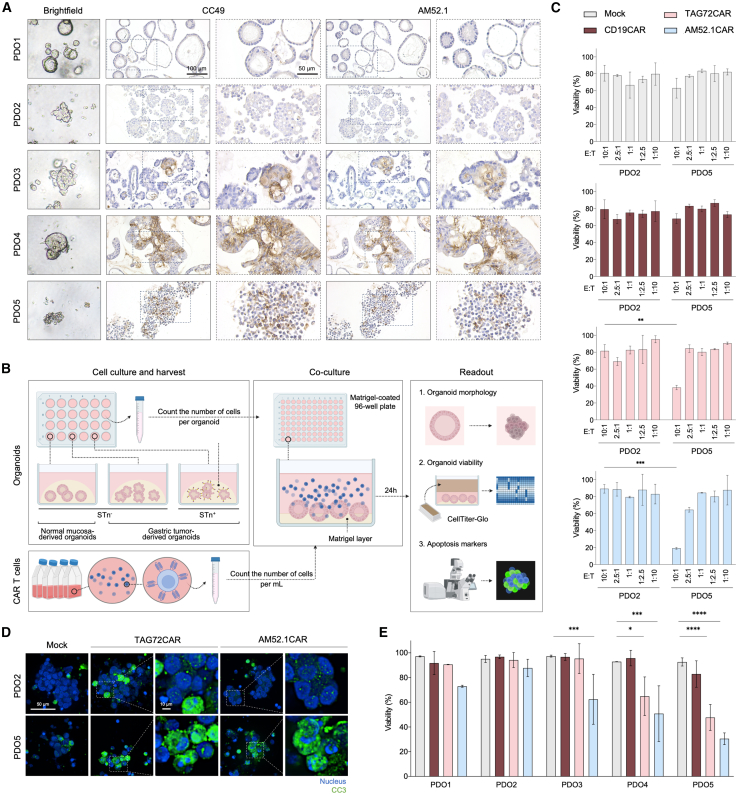
AM52.1CAR T cells are effective and specific in a preclinical model of patient-derived organoids (A) sialyl-Tn (STn) expression evaluation using CC49 and AM52.1 mAbs in five patient-derived organoids (PDOs). Scale bars correspond to 100 and 50 μm, as specified. (B) Schematic outlining the experimental design followed to establish the co-culture of the PDOs with the immune cells. (C) Viability of a STn-negative (PDO2) and a STn-positive (PDO5) PDOs upon co-culture with specified CAR T cells at different effector:target (E:T) ratios. Statistical comparison reflects differences at the same E:T ratio between the different organoids. *n* = 1 healthy donor. (D) Immunofluorescent analysis of cleaved caspase-3 (CC3) expression in the selected PDOs after co-culture with indicated CAR T cells at an E:T ratio of 10:1. Scale bars correspond to 50 and 10 μm, as specified. (E) Viability of PDOs derived from normal gastric mucosa (PDO1) and gastric tumor tissue (PDO2-5) with different levels of STn expression following co-culture with specified CAR T cells. The data correspond to the E:T ratio of 10:1. *n* = 3 healthy donors. Data are represented as mean ± standard deviation (SD). Statistical significance was determined using paired t test. ∗*p* ≤ 0.05, ∗∗*p* ≤ 0.01, ∗∗∗*p* ≤ 0.001, and ∗∗∗∗*p* ≤ 0.0001.

To evaluate CAR T cell functionality in a physiologically relevant setting, we further tested AM52.1CAR T cells against complex 3D cultures of GC PDOs. We used one organoid derived from normal gastric mucosa (PDO1), along with GC-derived PDOs from both STn-negative (PDO2) and STn-positive tumors with heterogeneous STn expression levels (PDO3-5) (Figure 4A). As depicted in Figures 4B, a co-culture assay of CAR T cells with gastric PDOs was performed, and organoid viability was assessed. Initially, a test with a STn-negative and a STn-positive PDO (PDO2 and 5, respectively) was conducted to determine the optimal E:T ratio for subsequent experiments. We observed that both AM52.1CAR T cells and TAG72CAR T cells effectively eliminated the PDO rich in STn expression at an E:T ratio of 10:1, while sparing the organoid lacking the target antigen (Figure 4C). These findings were corroborated by immunofluorescent detection of cleaved caspase-3 (CC3), which showed that incubation of the STn-positive PDO with AM52.1CAR T cells resulted in higher intracellular CC3 levels compared to those observed with the STn-negative PDO (Figure 4D). Notably, all GC-derived STn-expressing PDOs, even those with lower STn expression levels, were effectively killed by the AM52.1CAR T cells, contrasting with TAG72CAR T cells, which primarily affected highly STn-expressing PDOs (Figure 4E). Importantly, treatment with AM52.1CAR T cells had no impact on the organoid derived from normal gastric mucosa (Figure 4E). These observations were evident upon post-co-culture examination of the organoids under a light microscope, where AM52.1CAR T-treated STn-expressing GC PDOs displayed a noticeable reduction in cellular density and clear signs of apoptosis (Figure S4G). These data confirm that AM52.1CAR T cells are extremely potent and specific, even against structured targets.

### AM52.1CAR T cells effectively control sialyl-Tn-positive tumor growth in xenograft mouse models

**Figure 5:**
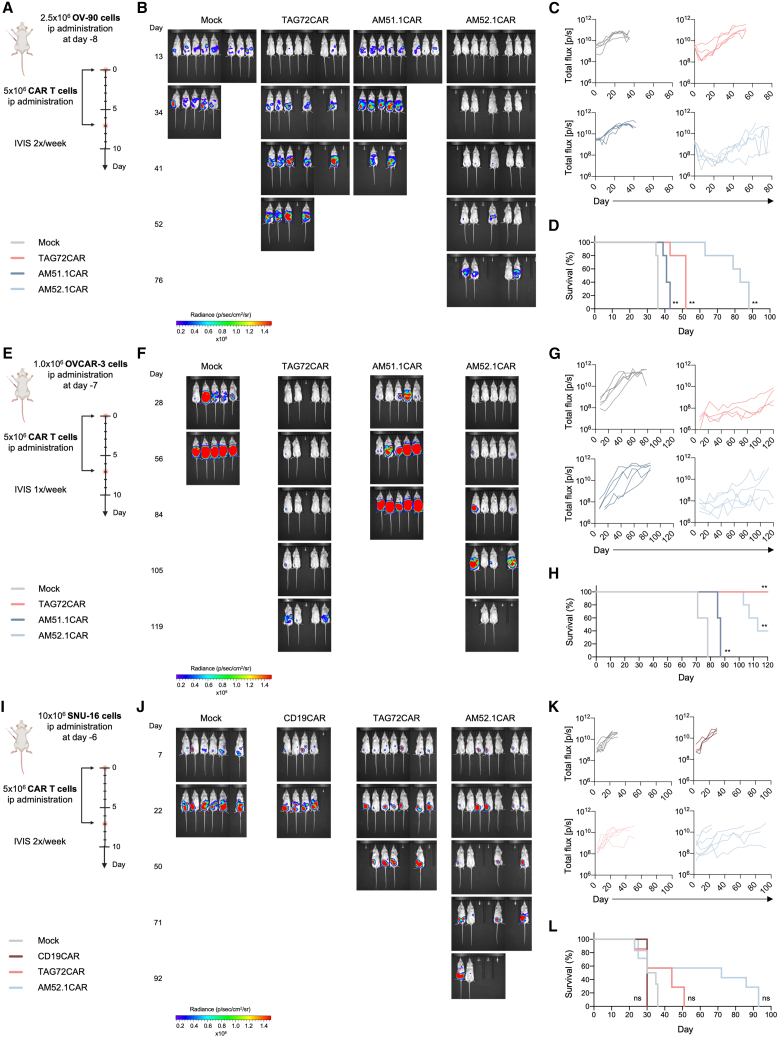
AM52.1CAR T cells efficiently control tumor growth in xenograft mouse models (A, E, and I) (A) OV-90, (E) OVCAR-3, and (I) SNU-16. Experimental scheme of the xenograft mouse models established. (B, F, and J) (B) OV-90, (F) OVCAR-3, and (J) SNU-16. Representative images of mice and bioluminescent signals at the indicated time points throughout the experiment. (C, G, and K) (C) OV-90, (G) OVCAR-3, and (K) SNU-16. Quantitative plots showing the bioluminescent signal (p/s) for each mouse over the course of the experiment. (D, H, and L) (D) OV-90, (H) OVCAR-3, and (L) SNU-16. Kaplan-Meier survival curves. Statistical analysis of survival curves was performed using the log rank (Mantel-Cox) test. ∗*p* ≤ 0.05 and ∗∗*p* ≤ 0.01.

We further investigated the efficacy of AM-derived CAR T cells in different xenograft mouse models. Three different aggressive human epithelial-derived cancer cell lines—OV-90, OVCAR-3, and SNU-16—were used to validate AM51.1- and AM52.1-based CAR T cells functionality. TAG72CAR T cells, along with CD19CAR T cells and Mock T cells, were used as positive and negative controls, respectively. Firstly, we tested the highly STn-positive OC cell line OV-90 (>80%, Figure 1E). Mice were intraperitoneally injected with the tumor cells as depicted in Figure 5A, randomized (Figure S5A), and treated with intraperitoneal (i.p.) administration of CAR T cells. Remarkably, AM52.1CAR T cells effectively controlled tumor growth (Figures 5B and 5C) and significantly improved survival (Figure 5D). In contrast, TAG72CAR T cells exhibited reduced efficacy in both tumor control and survival improvement. As expected, AM51.1CAR T cells had a minimal impact on tumor progression. Next, mice were intraperitoneally injected with the low STn-expressing OVCAR-3 OC cells (<20%, Figure 1E) as represented in Figure 5E, randomized (Figure S5B), and treated with i.p. administration of the different CAR T cell formulations. Both AM52.1CAR and TAG72CAR T cells demonstrated efficient tumor growth control (Figures 5F and 5G) and improved survival (Figure 5H). Notably, by the end of the experiment, two mice in the AM52.1CAR T-treated group and one mouse in the TAG72CAR T-treated group exhibited a very low tumor burden, albeit TAG72CAR T cells had a higher impact on survival than AM52.1CAR T cells. As previously observed, AM51.1CAR T cells only modestly reacted against the tumor. Thus, we excluded AM51.1CAR T cells for the last experiment and used CD19CAR T cells as an unspecific control. Lastly, SNU-16 cells (>40% STn^+^, Figure 1E) were injected intraperitoneally (Figure 5I), mice were randomized (Figure S5C), and subsequently treated with the CAR T cells. AM52.1CAR T cells showed strong tumor control (Figures 5J and 5K) and enhanced mice survival (Figure 5L). In comparison, TAG72CAR T cells demonstrated only moderate tumor growth inhibition and a less pronounced survival benefit. These findings underscore the therapeutic potential of AM52.1CAR T cells in targeting STn-positive cancer cells across varying expression levels. Importantly, none of the CAR constructs tested induced toxicity in animals, as evidenced by the stable weight during the course of the experiment (Figures S5D–S5F).

### AM52.1CAR T cells are robust enough to control a complex and diffuse solid tumor patient-derived xenograft model

**Figure 6:**
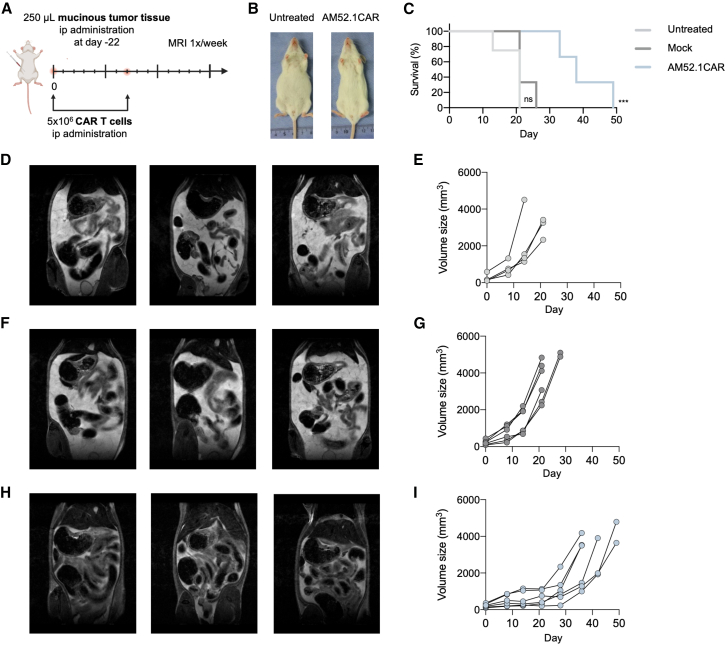
AM52.1CAR T cells control complex peritoneal metastases from colorectal cancer patient-derived xenograft model (A) Experimental scheme of the patient-derived xenograft (PDX) model established. (B) Images of two representative anesthetized mice 13 days post-treatment, either untreated or treated with AM52.1CAR T cells. (C) Kaplan-Meier survival curves for the PDX model. (D) Magnetic resonance imaging (MRI) images of three representative mice at week following CAR T cell injection. (E) Quantification of mucinous tissue in untreated mice (*n* = 4). (F) MRI images of three representative mice at week 2 post-injection of Mock T cells. (G) Quantification of mucinous tissue in Mock T cell-treated mice (*n* = 6). (H) MRI images of three representative mice at week 2 post-injection of AM52.1CAR T cell-treated mice. (I) Quantification of mucinous tissue in AM52.1CAR T cell-treated mice (*n* = 6). Statistical analysis of survival curves was performed using the log rank (Mantel-Cox) test. ∗*p* ≤ 0.05 and ∗∗*p* ≤ 0.01.

We additionally challenged the robustness of AM52.1CAR T cells in a PDX mouse model established from peritoneal metastases (PM) of CRC.^36^^,^^37^ A patient-derived PM-CRC sample, specifically mucinous tissue isolated from the peritoneal cavity, was stained by immunohistochemistry (IHC) with the mAb AM52.1. Expectedly, the secreted mucinous content was heavily sialylated and strongly stained by AM52.1 (Figure S6). The peritoneal mucinous carcinomatosis of patient 1, which exhibited a medium level of apical membrane staining with AM52.1 (Figure S6), was selected for investigating the *in vivo* efficacy of AM52.1CAR T cells. As depicted in Figure 6A, PM material was delivered intraperitoneally and, like observed in patients with PM, mice with disease progression exhibited visible accumulation of mucinous tissue in the peritoneal cavity (Figure 6B). Mice treated with AM52.1CAR T cells demonstrated a significantly higher survival benefit compared to both untreated and Mock T cell-treated mice (Figure 6C). Additionally, untreated mice (Figures 6D and 6E) and Mock T cell-treated mice (Figures 6F and 6G) displayed faster and larger accumulation of mucinous content than the AM52.1CAR T cell-treated group (Figures 6H and 6I). These results highlight the potency of AM52.1CAR T cells in a challenging preclinical *in vivo* model characterized by high heterogeneity and an unfavorable environment due to extensive mucin secretion.

## Discussion

In the current era of adoptive T cell immunotherapy, the meticulous selection of the target antigen stands out as the paramount determinant of success in cancer treatment. Tumor-associated carbohydrate antigens have emerged as appealing molecular targets for CAR T cell-based therapeutics due to their cell surface presentation and expression patterns highly restricted to malignant cells. Indeed, the truncated *O*-glycan STn antigen displays exclusive high expression in various tumors of epithelial origin and mostly absence in non-transformed healthy tissues.^15^^,^^16^^,^^17^^,^^18^^,^^19^ While subsequent advances and encouraging preclinical studies have been reported with STn-targeting CAR T cells, successful translation to the clinical setting has not yet been achieved.^27^

Previous STn-targeting CAR T cell approaches have employed various strategies to enhance both specificity and therapeutic efficacy. CARs targeting MUC1 glycoforms containing short *O*-glycan structures have demonstrated promising preclinical efficacy in leukemia and pancreatic cancer models.^38^ Modular platforms such as universal CARs, adapted from IgM antibodies recognizing both STn and α2,6-sialylated core 1 structures, have shown antitumor activity in bladder and breast cancer models.^29^^,^^30^ Additionally, a second-generation CC49-based CAR is currently undergoing evaluation for safety and tolerability in a phase 1 clinical trial in OC (NCT05225363).^28^ More recently, a fully human anti-STn CAR demonstrated preclinical efficacy in primary OC samples.^39^ Beyond traditional scFv-based CARs, alternative approaches, such as chimeric co-stimulatory receptors based on endogenous glycan-binding receptors such as sialic-acid-binding immunoglobulin-like lectin (Siglec)-9, present a viable and promising strategy for targeting aberrant glycosylation, including α2,6-sialylated structures.^40^

Our work introduces mAbs with unprecedented specificity, which were subsequently used as the basis for a CAR T cell therapy. We started by developing mAbs with restricted specificity by immunizing mice with a highly glycosylated mucin, OSM, known for its 98% STn content. Although eliciting a robust immune response toward glycans has proven to be a challenging task due to their characteristically low immunogenicity,^41^^,^^42^^,^^43^ our immunization strategy yielded a strong immune response, resulting in the generation of over 1,000 clones. Following an initial screening, two mAbs—AM51.1 and AM52.1—were selected. Notably, we were able to generate IgG/k antibodies, known for their highly specific binding, supporting a robust immune response against the target. ELISA-based screening uncovered specific binding to STn for the AM mAbs, contrarily to the well-established anti-STn CC49 mAb, which strongly reacted against non-sialylated OSM, in concordance to prior studies indicating CC49’s cross-reactivity with the GalNAc *per se*.^44^ A detailed glycan microarray analysis demonstrated AM52.1’s specific binding to STn, independently of the glycosylated amino acid and even at considerably low concentrations, whereas AM51.1 mainly recognized STn presented by serine residues with cross-reactivity with α2,6-linked sialic acids in poly-*N*-acetyllactosamine sequences. This observed binding pattern opens avenues for future research and applications aimed at elucidating the potential of AM51.1 to recognize this specific glycosylation signature. B72.3, tested in parallel as a control, demonstrated a clear preference for STn in serine over threonine, as previously reported by other studies,^26^ thus validating our analysis.

The specificity of the AM mAbs toward STn and their binding reliance on the sialic acid component were further confirmed in a cellular context using glycoengineered STn-enriched gastrointestinal cancer cell lines and endogenously STn-expressing gastric and tubo-ovarian adenocarcinoma cells. While the binding capacity of AM52.1 mirrored that of the benchmark B72.3 and CC49 mAbs, neuraminidase digestion revealed its higher sensitivity to the absence of sialic acids, demonstrating AM52.1’s higher specificity for the sialylated glycan structure compared to the existing antibodies. Surprisingly, AM51.1 showed minimal detection of naturally STn-expressing cells, suggesting that its binding may rely on a specific presentation of the glycan moieties. Despite being isolated from the same screen, AM51.1 and AM52.1 show quite different binding preferences, underscoring the critical need to validate glycan-directed antibodies through comprehensive library screening.

Given the major importance held by the accurate detection of STn in human samples for advancing cancer diagnostics and developing targeted therapies, the AM mAbs were tested against a diverse set of tumor tissues across multiple carcinoma types. Notably, AM52.1 exhibited selective staining of malignant tissues, similarly to the well-established B72.3 mAb. Considering that the success of antibody-based anti-cancer therapies relies on the ability of the selected mAbs to differentiate between cancerous and normal tissues, thus preventing on-target off-tumor toxicity,^45^^,^^46^ we additionally validated our AM antibodies using a panel of non-malignant tissues from healthy individuals. Importantly, both mAbs remained non-reactive toward healthy tissues, reinforcing their safety profile in a potential therapeutic context. While AM52.1 exhibited no reactivity toward PBMCs-derived B cells, monocytes, natural killer cells, and T cells, AM51.1 displayed reactivity against a significant subset of B cells from healthy donors. It is plausible that AM51.1’s binding to non-malignant B cells may be attributed to the presence of poly-*N*-acetyllactosamine sequences within this specific cell population.^47^

Due to the limited availability of CAR T cells for solid tumors, their unique glycome profiles, and the finely tuned specificity of the AM antibodies, we engineered AM mAbs-based scFvs for subsequent CAR generation. Interestingly, the sequence of the AM52.1 mAb had few somatic mutations, differing from the germline sequences identified on IMGT by only four amino acids. Initially, we conducted an *in silico* comparative analysis between these engineered scFvs and those derived from CC49, the most extensively explored anti-STn mAb in the CAR T field,^28^^,^^48^^,^^49^ uncovering some differences in both sequence and structure. Specifically, while AM52.1 exhibited higher sequence similarity to mCC49, structural comparisons surprisingly revealed a closer resemblance between the AM52.1-derived scFv and the huCC49-based counterpart, underscoring the complex relationship between sequence homology and structural similarity. Similarly, AM51.1 showed structural similarities to AM52.1 and mCC49, despite differences in sequence and binding specificity. The observed differences in sequence and structure among the compared mAbs, relative to their germline sequences, hint at very conserved and natural antibodies for which minimal variations may strongly impact the recognized epitope and specificity.^50^

Given the glycan-specificity held by the AM mAbs, second-generation CAR molecules have been designed based on their scFvs. Importantly, this CAR design has recently proven effective in redirecting T cells toward osteosarcoma.^51^ Initial validation confirmed that both AM-derived CARs were efficiently expressed at the T cell membrane, non-toxic, active, and specific. However, comprehensive *in vitro* functional analyses, including degranulation, target cell killing, and cytokine secretion, revealed that only AM52.1CAR T cells effectively engaged and responded to STn-expressing cancer cell lines. AM52.1CAR T cells upregulated the degranulation marker CD107a, induced robust target cell lysis, and secreted key pro-inflammatory cytokines such as TNF-α, IFN-γ, IL-13, IL-8, and MIP-1β, which play crucial roles in CAR T cell effector functions, including recruitment of innate immune cells, enhancement of T cell proliferation and persistence, and direct cytotoxic effects. Importantly, AM52.1CAR T cells secreted cytokines even in response to targets with relatively low STn expression, such as OVCAR-3, underscoring their potential therapeutic value in eliminating tumors with low antigen expression. In contrast, AM51.1CAR T cells failed to respond to STn-positive targets, in line with the reduced immunoreactivity observed for AM51.1. While antibody specificity does not always predict CAR activity, our data clearly indicate that AM51.1-based CARs are functionally inert in this context. Importantly, and unlike the benchmark huCC49-derived TAG72CAR, AM52.1CAR T cells did not react to STn-negative cancer or normal cell lines, including primary fibroblasts and endothelial cells of lung and umbilical vein origin, demonstrating a tighter specificity and reinforcing their safety advantage. These findings collectively underscore the therapeutic promise of AM52.1CAR T cells for treating solid tumors characterized by heterogeneous STn expression.

Using gastric PDOs as a preclinical model, we showcased the efficacy of AM52.1CAR T cells in eradicating GC PDOs expressing varying levels of STn, underscoring their potential therapeutic value in eliminating tumors, including those with low expression of the target antigen. Once more, AM52.1CAR T cells proved more effective than TAG72CAR T cells, which efficacy was limited to GC PDOs displaying high STn content. Importantly, AM52.1CAR Ts exhibited no reactivity against normal gastric mucosa-derived organoids, emphasizing once again their favorable safety profile.

In OV-90, OVCAR-3, and SNU-16 xenograft mouse models, CAR T cells were administrated intraperitoneally to mimic locoregional delivery,^52^ a promising approach in solid tumors’ treatment also being explored in a clinical trial with TAG72CAR T cells (NCT05225363). AM52.1CAR T cells demonstrated significant efficacy in controlling OC and GC progression, outperforming TAG72CAR T cells in OV-90- and SNU-16-derived models. Interestingly, despite CC49 showing higher binding to SNU-16 cells compared to AM52.1 mAb, this did not translate into superior CAR T cell activity, further highlighting that the binding profile of an antibody does not always directly translate into CAR functionality. Despite low STn expression by OVCAR-3 cells, treatment of OVCAR-3 xenograft mice with either AM52.1CAR T cells or TAG72CAR T cells significantly prolonged their survival up to the 120-day humane endpoint. The dynamic interplay of STn with the TME and the *in vivo* selection pressure may contribute to the observed results, as STn has been linked to immune evasion mechanisms in cancer cells.^53^^,^^54^ The observed discrepancy suggests a dynamic rather than static expression of heavily sialylated transmembrane proteins in OVCAR-3 cells, similar to the increases in STn expression observed in endometrial cells during the menstrual cycle, hinting at the biological implications of STn.^55^ In the OVCAR-3-derived model, TAG72CAR T cells demonstrated higher potency compared to AM52.1CAR T cells, consistent with CC49 staining persistence even after neuraminidase digestion of OVCAR-3 cells, suggesting potential cross-reactivity with an additional antigen, such as Tn. Regarding AM51.1CAR T cells, despite their poor *in vitro* functional performance, *in vivo* studies indicated that AM51.1-derived CAR T cells could exert a modest impact on tumor growth. Notably, no significant weight loss or clinical signs of toxicity were observed in any treatment group throughout all *in vivo* experiments, further supporting the tolerability and safety of AM52.1CAR T cells in these models.

An additional demonstration of the clinical relevance of AM52CAR Ts cells was provided through a challenging PM-CRC PDX mouse model. This model closely recapitulates an aggressive and immune-restricted TME enriched with heavily sialylated mucins and characterized by a highly viscous peritoneal milieu, factors that are known to impede CAR T cell trafficking and function. Given the well-established role of STn in promoting immune evasion and contributing to an immunosuppressive TME, effective CAR T cell therapy in such conditions is particularly challenging.^53^^,^^54^ Despite these unfavorable features, locoregional administration of AM52.1CAR T cells achieved impressive tumor growth control and significantly improved survival, supporting their potential as a treatment approach for rare cancers.

In conclusion, we have successfully generated two mAbs, namely AM51.1 and AM52.1. While AM51.1 demonstrates dual recognition on *O*-glycans of STn and α2,6-sialylated poly-*N*-acetyllactosamine structures, AM52.1 excels in its precise targeting of the cancer-associated STn antigen, overcoming a key limitation of previous anti-STn antibodies that often displayed cross-reactivity with structurally related glycans. We anticipate that AM52.1 can address specific gaps in the existing landscape of anti-STn antibodies for diagnostic, but mainly for therapeutic applications, as its refined specificity enables safer and more selective cancer targeting. Based on their clinical potential in cancer immunotherapies, owing to their selectivity for STn-expressing cancers and an improved safety profile, we have engineered AM mAbs-based CAR T cells. Notably, AM52.1CAR T cells exhibited robust functionality against different solid tumors, both *in vitro* and *in vivo*. Considering STn’s contribution to immune evasion, future work could improve therapeutic outcomes by combining AM52.1CAR T cells with checkpoint inhibitors or agents that modulate the TME. Furthermore, detailed profiling of the immune landscape of tumors with suboptimal responses to AM52.1CAR T cells could identify predictive biomarkers to guide personalized treatment strategies. Altogether, our study introduces a CAR T cell therapy with high specificity, potent anti-tumor activity, and strong safety profile. We propose the application of AM52.1CAR T cells as a promising and effective therapeutic approach for STn-expressing epithelial-derived solid tumors, paving the way for their clinical translation.

### Limitations of the study

Our findings support the therapeutic potential of AM52.1CAR T cells, yet some limitations should be acknowledged. First, while the AM antibodies effectively detected STn across multiple carcinomas, we did not explore potential correlations with clinicopathological variables such as age, sex, tumor subtype, grade, or neoadjuvant therapy. Larger, clinically annotated cohorts are required to assess the predictive value of AM51.1 and AM52.1. In addition, although we compared AM51.1 and AM52.1 to established anti-STn antibodies such as B72.3 and CC49, given their relevance in diagnostics and in the CAR T cell field, broader benchmarking against other currently available antibodies could provide deeper insight into relative specificity. A key consideration is antigen heterogeneity and the potential for immune escape. Although AM52.1CAR T cells exhibited efficacy even against tumors with low STn density, we did not directly investigate mechanisms such as antigen loss or glycan remodeling, which could compromise treatment durability. Future studies employing longitudinal sampling will be essential to uncover these escape pathways and refine target selection, potentially informing strategies such as dual CAR targeting and/or adapting the treatment administration approach. The translational relevance of our *in vivo* models also presents limitations. All experiments were performed in female immunodeficient mice using i.p. delivery, a clinically relevant approach for peritoneal disease but insufficient to model systemic trafficking, human immune interactions, or cytokine-related toxicities. Sex-independent evaluation in humanized mouse models, non-human primates, or early clinical trials will be needed to better predict potential sex-related variability, persistence, biodistribution, and safety in patients. Regarding safety, we observed no signs of acute toxicity *in vitro* or *in vivo*. AM52.1 showed no binding to normal human tissues or healthy donor immune cells, and no off-tumor effects were detected in mice. Nonetheless, long-term safety remains to be established. Toxicology studies in non-human primates or early clinical trials will be critical to exclude delayed or tissue-specific adverse events. Finally, we did not investigate therapeutic combinations that could further improve efficacy. Given STn’s role in immune evasion, combining AM52.1CAR T cells with immune checkpoint inhibitors or TME-modulating agents could enhance clinical benefit. Additionally, profiling the immune landscape of non-responsive tumors may uncover predictive biomarkers to guide personalized CAR T cell strategies.

## Resource availability

### Lead contact

Further information and requests for resources and reagents should be directed to and will be fulfilled by the lead contact, Sébastien Wälchli (sebastw@ous-hf.no).

### Materials availability

All unique materials generated in this study, including the AM52.1 mAb and AM52.1CAR vectors, will be available upon request. Requests may be subjected to a payment and/or a completed material transfer agreement, particularly if there is potential for commercial application.

### Data and code availability

• Raw data reported in this paper will be shared by the lead contact upon request.
• This paper does not report original code.
• Any additional information required to reanalyze the data reported in this paper is available from the lead contact upon request.

## Acknowledgments

The authors thank Åge Winje Brustad from the OUH for the assistance with the hybridoma production. The authors are grateful to Professor Ian Mackenzie from the Queen Mary University of London for generously providing the Ca1 cell line. We acknowledge the support of the i3S Scientific Platforms, particularly the Histology and Electron Microscopy, the Translational Cytometry, and the BioSciences Screening facilities. Additionally, the authors acknowledge the “P.CCC: Centro Compreensivo de Cancro do Porto”—NORTE-01-0145-FEDER-072678, supported by Norte Portugal Regional Operational Program (NORTE 2020), under the PORTUGAL 2020 Partnership Agreement, through the European Regional Development Fund (ERDF). We thank the Flow Cytometry Core Facility and the Department of Comparative Medicine at OUH. The authors thank Wafa Kefi, Emilie Einersten, and Karoline Strømmen Martinsen (OUH) for PBMC preparation. The authors are grateful to Professor Gunhild Mari Mælandsmo from OUH for kindly providing the HM-8 and HM-29 cell lines. Finally, we are grateful to the Radiumhospitalets legater and the OvaCure foundation (Denmark) for their support. This research was funded by the Portuguese Foundation for Science and Technology (FCT) (PTDC/MEC-ONC/0491/2021 to C.A.R., 2022.04678.CEECIND to C.G., 2022.02109.CEECIND to F.P., 2020.05483.BD to R.A., 2021.05495.BD to L.S.-F., and UI/BD/150829/2021 to A.F.C.). This work was also funded by the Bilateral Relations—EEA Grants Portugal (NOVEL-CAR Project). C.F. was a PhD fellow of the National Centre for Research and Development within POLNOR program NOR/POLNOR/ALTERCAR/0056/2019. S.W. received funding from the Norwegian Cancer Society (IIDEA, no. 223171), the Norwegian Childhood Cancer Society (PERCAP, no. 230004), the Research Council of Norway (nos. 337468, 351914, and 351915), and the Norwegian Health Authority South-East (nos. 2024080 and 2022009 for K.K. PhD). E.M.I. received founding from the Research Council of Norway through a KSP-2021 (CellFit, no. 326811). V.G. was supported by the Leona M. and Harry B. Helmsley Charitable Trust (no. 2019PG-T1D011), the UiO World-Leading Research Community, UiO: LifeScience Convergence Environment Immunolingo, EU Horizon 2020 iReceptorplus (no. 825821), the Norwegian Cancer Society Grant (no. 215817), the Research Council of Norway projects (nos. 300740, 311341, and 331890), IKTPLUSS project (no. 311341), and the European Union (ERC, AB-AG-INTERACT, 101125630). This project has also received funding from the European Union’s Horizon 2020 research and innovation program under the Marie Skłodowska-Curie grant agreement no 801133 (to P.R.). This study was partially supported by the Research Council of Norway through a FRIPRO project (AMIDE, no. 326300), The Norwegian Cancer Society project (IIDEA, no. 223171), and Norwegian Childhood Cancer Society projects (PICCA2, PERCAP, and Ped-HemaCAR) to E.M.

## Author contributions

R.A., conceptualization, methodology, investigation, data analysis, writing – original draft, and writing – review & editing; C.F., conceptualization, methodology, investigation, data analysis, and writing – review & editing; D.J.W., methodology and writing – review & editing; L.S.-F., methodology and writing – review & editing; K.G.F., methodology and writing – review & editing; E.S., methodology and writing – review & editing; A.F.C., methodology and writing – review & editing; K.K.,: methodology and writing – review & editing; R.H., formal analysis and writing – review & editing; A.M.L., methodology and writing – review; P.R., methodology and writing – review & editing; P.G., methodology and writing – review & editing; E.M., methodology and writing – review & editing; L.B., methodology and writing – review & editing; B.D., methodology and writing – review & editing; V.G., methodology and writing – review & editing; D.E.C., methodology and writing – review & editing; F.P., methodology and writing – review & editing; K.F., methodology and writing – review & editing; C.G., conceptualization, supervision, and writing – review & editing; E.M.I., conceptualization, funding acquisition, supervision, and writing – review & editing; C.A.R., conceptualization, funding acquisition, supervision, and writing – review & editing; S.W., conceptualization, funding acquisition, supervision, and writing – review & editing.

## Declaration of interests

V.G. declares advisory board positions in aiNET GmbH, Enpicom B.V, Absci, Omniscope, and Diagonal Therapeutics. V.G. is a consultant for Adaptyv Biosystems, Specifica Inc., Roche/Genentech, Immunai, Proteinea, LabGenius, and FairJourney Biologics.

## STAR★Methods

### Key resources table

**Table:** 

REAGENT or RESOURCE	SOURCE	IDENTIFIER
**Antibodies**		

α-STn (B72.3)	Santa Cruz Biotechnology	Cat#sc-20042; RRID: N/A
α-STn (CC49)	Santa Cruz Biotechnology	Cat#sc-20043; RRID: N/A
α-STn (AM51.1)	This paper	N/A
α-STn (AM52.1)	This paper	N/A
α-Tn (1E3)	Clausen and Hakomori, unpublished	N/A
α-β-Actin (C4)	Santa Cruz Biotechnology	Cat#sc-47778; RRID: AB_626632
APC-conjugated α-human CD34 (4H11)	Thermo Fisher Scientific	Cat#17-0349-42; RRID: AB_2016672
Biotinylated goat α-mouse IgG (Fab specific)	Jackson Immunoresearch	Cat#115-035-003; RRID: AB_10015289
Mouse α-human CD3 (OKT)	Thermo Fisher Scientific	Cat#MA1-10175; RRID: AB_11157849
Mouse α-human CD28 (CD28.6)	Thermo Fisher Scientific	Cat#16-0288-81; RRID: AB_468924
PE-Cy5-conjugated α-CD107a	BD Biosciences	Cat#555802; RRID: AB_396136
BV421-conjugated α-CD4	Biolegend	Cat#317434; RRID: AB_11150413
BV711-conjugated α-CD8	BD Biosciences	Cat#563677; RRID: AB_274446
α-CC3	Cell Signaling Technology	Cat#9661; RRID: AB_2341188
HRP-conjugated rabbit α-mouse IgG	Dako	Cat#P0260; RRID: AB_2636929
HRP-conjugated goat α-mouse IgG	Jackson Immunoresearch	Cat#115-035-003; RRID: AB_10015289
AlexaFluor™ 555-conjugated goat α-mouse IgG	Thermo Fisher Scientific	Cat#A21422; RRID: AB_2535844
Alexa Fluor™ 488-conjugated goat α-mouse IgG	Thermo Fisher Scientific	Cat#A28175; RRID: AB_2536161
Alexa Fluor™ 594-conjugated goat α-mouse IgG	Thermo Fisher Scientific	Cat#A11032; RRID: AB_2534091
Biotinylated rabbit α-mouse IgG	Dako	Cat#E0354; RRID: AB_2687571
Streptavidin-PE	Biolegend	Cat#405203; RRID: N/A

**Bacterial and virus strains**		

NEB 5-alpha competent *E. coli*	New England Biolabs	Cat#C2987H

**Biological samples**		

Peripheral blood mononuclear cells (PBMCs)	Oslo University Hospital	N/A
Patient-derived organoids (PDOs)	Unidade Local de Saúde de São João	N/A
Patient-derived xenografts (PDXs)	Oslo University Hospital	N/A
Carcinoma human tissues	Unidade Local de Saúde de São João and Portuguese Oncology Institute of Porto	N/A
Healthy human tissues	US Biomax	Cat#0131-FDA999-L942

**Chemicals, peptides, and recombinant proteins**		

Fetal bovine serum (FBS)	Gibco	Cat#10500-064
HyClone FBS	GE Healthcare Life Sciences	Cat#SH30071.03
Human serum (HS)	PAN-Biotech GmbH	Cat#P40-2702HI
Recombinant human IL-2	Thermo Fisher Scientific	Cat#200-02
Lysis Buffer 17	R&D Systems	Cat#AR010
PhosSTOP	Roche	Cat#4906845001
cOmplete protease inhibitor cocktail	Roche	Cat#04693132001
Retronectin	Takara Bio	Cat#T100B
*Clostridium perfringens*	Sigma	Cat#11585886001
GolgiStop	BD Biosciences	Cat#554724
GolgiPlug	BD Biosciences	Cat#555029
D-Luciferin	PerkinElmer	Cat#122799
Paraformaldehyde (PFA)	Thermo Fisher Scientific	Cat#43368

**Critical commercial assays**		

DC protein assay kit	Bio-Rad	Cat#500-0111
Bio-Plex Pro Human Cytokine 17-plex assay	Bio-Rad	Cat#M5000031YV
PowerPlex 16 HS System kit	Promega	Cat#DC2101

**Experimental models: Cell lines**		

J-76	Mirjam Heemskerk	Heemskerk et al.^56^
Ca1	IC Mackenzie	Mackenzie^57^
HM-29	Gunhild Mari Mælandsmo	Svendsen et al.^58^
HM-8	Gunhild Mari Mælandsmo	Svendsen et al.^58^
Hek-293-Phoenix	LCG Genomics GmbH	ATCC-CRL-3213
MKN45	Japanese Cancer Research Bank	RCB1001
BJ	American Type Culture Collection	CRL-2522
BL-41	Leibniz Institute DSMZ-German Collection	ACC 160
Caov-3	American Type Culture Collection	HTB-75
CCD-18Co	American Type Culture Collection	CRL-1459
COLO205	American Type Culture Collection	CCL-222
HCT-116	American Type Culture Collection	CCL-247
HULEC-5a	American Type Culture Collection	CRL-3244
HUV-EC-C	American Type Culture Collection	CRL-1730
LS174T	American Type Culture Collection	CL-188
MRC-5	American Type Culture Collection	CCL-171
NCI-N87	American Type Culture Collection	CRL-5822
OSA (SJSA-1)	American Type Culture Collection	CRL-2098
OV-90	American Type Culture Collection	CRL-3585
OVCAR-3	American Type Culture Collection	HTB-161
PC-3	American Type Culture Collection	CRL-1435
SK-MEL-5	American Type Culture Collection	HTB-70
SNU-16	American Type Culture Collection	CRL-5974
U-251	Anna Golebiewska	Torsvik et al.^59^

**Experimental models: Organisms/strains**		

NOD-*Prkdc*^*scid*^*-IL2rg*^*Tm1*^/Rj (NXG) mice	Janvier Labs	NXG

**Software and algorithms**		

GraphPad Prism v9.0	GraphPad Software	https://www.graphpad.com/
FlowJo 10.7.1	BD Biosciences	https://www.flowjo.com/
BioRender	BioRender	https://www.biorender.com/
R 4.2.0	R Core Team	https://www.r-project.org/
RStudio 2022.07.1 + 554	Posit	https://posit.co/products/open-source/rstudio/
IVIS-200 imaging system	Perkin Elmer	https://www.perkinelmer.com/

### Experimental model and study participant details

#### Cell lines and culture conditions

The human GC cell line MKN45 was obtained from the Japanese Cancer Research Bank (Tsukuba, Japan), and the human embryonic kidney cell line HEK-293-Phoenix was obtained from LCG Genomics (LGC Genomics GmbH, Berlin, Germany). All other cell lines were purchased from the American Type Culture Collection (ATCC; Manassas, VA, USA), unless otherwise specified. The malignant oral squamous cell carcinoma cell line Ca1 was generously provided by Professor IC Mackenzie (Queen Mary University of London, London, UK).^57^ The melanoma cell lines HM-8 and HM-29, established from melanoma patients as previously described,^58^ were kindly provided by Professor Gunhild Mari Mælandsmo (Oslo University Hospital (OUH), Oslo, NO), with use approved by the Norwegian Research Ethics Committee (approval no. 2011/2183). The Jurkat-derived J-76 cells, an immortalized human T lymphocyte line originating from a patient with acute T cell leukemia, was a kind gift from Professor Mirjam Heemskerk (Leiden University Medical Center, Leiden, Netherlands).

All cell lines were cultured at 37°C in a humidified atmosphere of 5% CO_2_. The following media were used: Roswell Park Memorial Institute (RPMI) 1640 (Gibco, Waltham, MA, USA) for MKN45, SNU-16, NCI-N87, BL-41, OSA, HCT-116, COLO205, OV-90, OVCAR-3, and J-76; Dulbecoo’s Modified Eagle Medium (DMEM; Gibco) for LS174T, Caov-3, and HEK-293-Phoenix; Eagle’s Minimum Essential Medium (EMEM; ATCC) for CCD-18Co, SK-MEL-5, BJ, MRC-5, U-251; and DMEM/F12 (Thermo Fisher) for PC-3, HUV-EC-C, and HULEC-5a. Media were supplemented with 10% fetal bovine serum (FBS; Gibco), except for the HEK-293-Phoenix cells, which were maintained with 10% HyClone FBS (GE Healthcare Life Sciences, Logan, UT, USA), and HUV-EC-C and HULEC-5a cells, which were cultured with 20% FBS (Gibco).

All cell lines had their identity confirmed by short tandem repeat profiling using the PowerPlex 16 Human serum (HS) System kit (Promega, Madison, WI, USA), and were routinely tested for mycoplasma contamination by PCR amplification.

The MKN45, COLO205, and LS174T cell lines were genetically modified by CRISPR/Cas9 methodology as previously described^60^ to express the STn and Tn glycan structures, by knocking-out the expression of the C1GalT1 enzyme.

#### Human samples

Primary gastric (*n* = 4), colon (*n* = 5), pancreatic (*n* = 5), prostate (*n* = 5), and tubo-ovarian (*n* = 5) carcinoma specimens were obtained from the archives of the Unidade Local de Saúde de São João (ULSSJ), Porto, Portugal and from the Portuguese Oncology Institute of Porto (IPO Porto), Portugal. Tissue samples from healthy individuals were obtained from US Biomax, Rockville, MD, USA. Patients ranged in age from 34 to 86 years, with 56% females and 44% males. The collection and handling of human samples were performed in accordance with the national regulatory law governing the use of biological specimens. This process was conducted in strict compliance with ethical guidelines which required written informed consent from the patients after the approval by the local institutional ethical committee. Clinicopathological information was obtained from patients’ clinical records. For IHC analysis, formalin-fixed paraffin-embedded tissue sections were used.

Human gastric organoids were previously established from fresh tissue samples collected at ULSSJ (ethical approved reference number CES 223/2021).^61^ The selected organoids included one derived from normal gastric mucosa of a non-tumoral obese donor and four derived from GC tumor tissues, obtained from patients aged between 24 and 88 years, including 3 females and 2 males.

PDX material was obtained from a 68-year-old woman with PM-CRC patients undergoing cytoreductive surgery with hyperthermic i.p. chemotherapy (CRS-HIPEC) at the OUH, Norway.^36^^,^^62^

Buffy coats from healthy donors aged 18 to 60 years were purchased from the blood bank of the OUH, with approval from the Regional Committee for Medical Research Ethics South-East (2019/121). Human PBMCs were then isolated by density gradient and cryopreserved in liquid nitrogen until use.

#### Animal models

Female non-obese diabetic (NOD)-*Prkdc*^*scid*^ *-IL2rg*^*Tm1*^/Rj (NXG) mice were bred in-house and maintained in a strictly controlled hygienic and pathogen-free facility, in accordance with an approved institutional animal care protocol. Study and control animals were housed in the same room under 12-h light-dark cycles, with humidity levels between 30 and 70% and ambient temperature of 20°C–26°C. Health status was monitored regularly based on clinical parameters such as posture, activity level, fur condition, skin health, and weight loss. Humane endpoint criteria included the following indicators: (i) weight loss equal to or greater than 20% from the baseline, (ii) abnormal gait, paralysis, or difficulty in normal ambulation, (iii) respiratory distress, (iv) lethargy or prolonged recumbence, (v) abnormal neurological behaviors, (vi) abnormal abdominal distention, or (vii) within 120 days after the start of the experiment. Mice reaching humane endpoints were euthanized by cervical dislocation. All animal experiments conducted in this study received approval from the Norwegian Food Safety Authority (FOTS ID 11118).

### Method details

#### Generation of monoclonal antibodies

##### Immunization and hybridoma development

Monoclonal antibody production was carried out essentially as described previously.^63^ Female Balb/c mice were primed subcutaneously with complete Freund’s adjuvant (Sigma-Aldrich, St. Louis, USA) containing 20 μg of purified OSM.^64^ Two subsequent booster immunizations were performed one and three months later, administering 30 μg of OSM subcutaneously, emulsified in Freund’s incomplete adjuvant. Two months following the final booster dose, a single ip injection of 50 μg of antigen in saline was given 4 days before fusion. Hybridomas were produced by polyethylene glycol (PEG)-facilitated fusion of splenocytes to the NS0 myeloma cell line. Hybridomas were screened for mAbs using an antibody-capture assay. In brief, supernatants from ten 96-well microplate cultures were replica-plated into two sets of 96-well MaxiSorp plates (Thermo Fisher Scientific, Waltham, MA, USA) coated with either OSM or neuraminidase-treated OSM, A-OSM. Antibody binding to the solid phases was subsequently assessed using a europium-labelled sheep anti-mouse IgG tracer and time-resolved fluorimetry. Hybridomas producing mAbs with strong binding to OSM and minimal reactivity to A-OSM were selected for further characterization.

#### Characterization of mAbs

##### Antibodies

All antibodies used in the assays described below are listed in the key resources table.

##### Enzyme-linked immunosorbent assay

Enzyme-linked immunosorbent assay (ELISA) was performed in 96-well MaxiSorp plates (Thermo Fisher Scientific) coated overnight (ON) at 4°C with 100 μL of OSM, in eight 2-fold serial dilutions in coating buffer (50 mM sodium carbonate/bicarbonate pH 9.5), starting with 0.1 μg/mL. A-OSM was used as negative control. After three washing steps with wash buffer (0.05% Tween/phosphate-buffered saline (PBS)), blocking was performed with 150 μL of blocking solution (1% bovine serum albumin (BSA)/PBS) for 1h at room temperature (RT), followed by incubation with 50 μL of mAbs diluted in blocking solution (B72.3 at 1 μg/mL, CC49, AM51.1, and AM52.1 at 5 μg/mL, and 1E3 at 1:2 dilution) for 2h at RT. Following washing steps, bound antibodies were detected with 50 μL of horseradish peroxidase (HRP)-conjugated rabbit anti-mouse immunoglobulins (0.3 μg/mL; Dako, Berlin, Germany) for 1h at RT. After three additional washing steps, 50 μL of 3,3′,5,5′-tetramethylbenzidine (TMB; Thermo Fisher Scientific) substrate were added to each well, plates were incubated in the dark, and the reaction was stopped by adding 50 μL of 0.5 M HCl. Optical Density was measured at 450 nm on a microplate reader (BioTek *Synergy MX Microplate Reader, Winooski, VT, USA).*

##### Glycan microarray

*G*lycan microarrays (Z-Biotech, Aurora, CO, USA) composed of 94 serine- or threonine-tagged *O*-glycans were used according to the manufacturer’s instructions. Glycans were immobilized onto the microarray substrates in triplicate. Mouse IgG was spotted and used as a positive control. Briefly, slides were blocked with Glycan Array Blocking Buffer at RT for 30 min. Following the blocking treatment, primary antibodies were added (10, 2, or 0.4 μg/mL) and incubated at RT for 1h. After washing steps, Alexa Fluor 555-conjugated anti-mouse IgG antibody (10 μg/mL; Thermo Fisher Scientific) was incubated for 1h at RT. Slides were washed, dried, and then scanned with a InnoScan 710 scanner (Innopsys, Carbonne, France). Fluorescence intensities were measured, and background was subtracted using Mapix software (Innopsys).

##### Flow cytometry

Binding of AM antibodies to cell surface STn was determined through standard flow cytometric staining. Briefly, subconfluent cell cultures (10^5^ cells/antigen) were detached with accutase (Gibco) or trypsin-EDTA (Gibco), and stained with the primary antibodies (B72.3, CC49, AM51.1, and AM52.1 at 3 μg/mL, and 1E3 at 1:2 dilution) for 30 min at 4°C or 15 min at RT. Cells were then labeled with secondary anti-mouse Alexa Fluor 488- or Alexa Fluor 594-conjugated antibody (5 and 10 μg/mL, respectively; Thermo Fisher Scientific) for 30 min at 4°C or 15 min at RT. Cells were strained, labeled with 4′,6-diamino-2-phenylindole dihydrochloride (DAPI; Sigma-Aldrich, St. Louis, MO, USA), and measured using a BD FACSCanto II instrument (BD Biosciences, San Jose, CA, USA) or a BD LSRFortessa (BD Biosciences). Data were analyzed using the FlowJo software (BD Biosciences).

##### Immunofluorescence

Subconfluent cells growing on glass coverslips (Marienfeld Superior, Lauda-Königshofen, Germany) were fixed with 4% paraformaldehyde (PFA; Thermo Fisher Scientific) and permeabilized with 0.5% Triton X-100 (Sigma-Aldrich) for 10 min at RT. After blocking with UltraVision Protein Block (Thermo Fisher Scientific) for 5 min at RT, incubation with the primary antibodies (B72.3, CC49, AM51.1, and AM52.1 at 5 μg/mL, and 1E3 at 1:2 dilution) was performed ON at 4°C. Cells were labeled with Alexa Fluor 488- or Alexa Fluor 594-conjugated secondary antibody (2 and 4 μg/mL, respectively; Thermo Fisher Scientific) for 1h at RT, and nuclear staining with DAPI (Sigma-Aldrich). Slides were mounted in VectaShield (Vector Laboratories, Burlingame, CA, USA). Images were acquired with a Zeiss Axiocam MR apparatus, using AxioVision v4.8 software (Carl Zeiss MicroImaging GmbH, Jena, Germany).

##### Western blotting

Subconfluent cell cultures were washed twice with ice-cold PBS and protein lysates were directly collected by scrapping cells in Lysis Buffer 17 (R&D Systems, Minneapolis, MN, USA) supplemented with 10% PhosSTOP (Roche, Basel, Switzerland) and 4% cOmplete protease inhibitor cocktail (Roche). Total protein concentrations of whole cell lysates were determined using the DC protein assay kit (Bio-Rad, Hercules, CA, USA). Equal amounts of protein were separated by polyacrylamide gel electrophoresis (SDS-PAGE) and blotted onto nitrocellulose membranes (GE Healthcare, Chicago, IL, USA). For glycan detection, membranes were blocked with 5% BSA in PBS containing 0.1% Tween (PBS-T) for 1h at RT, followed by ON incubation with the primary antibodies (B72.3, AM51.1, and AM52.1 at 5 μg/mL, and 1E3 at 1:2 dilution) at 4°C, and 1h incubation at RT with HRP-conjugated polyclonal anti-mouse (0.16 μg/mL; Jackson Immunoresearch, Cambridgeshire, United Kingdom) diluted in 5% and 1% BSA/PBS-T, respectively. Specific signals were detected using the enhanced chemiluminescence (ECL) detection method. Anti-β-Actin (0.04 μg/mL; Santa Cruz Biotechnology, Santa Cruz, CA, USA) was used as loading control.

##### Neuraminidase treatment

Sialic acid removal was achieved by treatment with *Clostridium perfringens* neuraminidase (Sigma). Briefly, cells (10^7^/condition) were incubated with 0.5 U/mL of neuraminidase diluted in 200 μL of plain media for 90 min at 37°C for the flow cytometry analysis. For immunofluorescent detection, treatment of cells with 0.1 U/mL of neuraminidase diluted in 0.1 M sodium acetate buffer (pH 5.5) for 2h at 37°C allowed the removal of sialic acids. For Western blotting analysis, sialic acid removal was achieved by treating blotted nitrocellulose membranes with 0.1 U/mL of the sialidase diluted in 0.1 M sodium acetate buffer (pH 5.5) for 2h at 37°C.

##### Immunohistochemistry

The binding specificity of AM mAbs toward STn epitope was analyzed in primary gastric, colorectal, pancreatic, prostate, and tubo-ovarian carcinoma tissue sections, and in a series of normal healthy tissues. Briefly, deparaffinization and rehydration of tissue sections was performed, followed by neutralization of endogenous peroxidase activity with 3% hydrogen peroxide (H_2_O_2_) in methanol. For blocking of unspecific reactivity, the UltraVision Protein Block (Thermo Fisher Scientific) was used for 5 min at RT. Tissue sections were then incubated ON at 4°C with the primary antibodies diluted in 5% BSA/PBS (3 μg/mL), followed by a 30-min incubation at RT with a goat anti-mouse biotin-labeled secondary antibody (6 μg/mL; Dako). Signal amplification was achieved using an avidin-biotin complex kit (Vector Laboratories) for 30 min at RT. Tissue sections underwent chromogenic staining with 3,3′-diaminobenzidine (DAB, Sigma-Aldrich) previously activated with 0.01% H_2_O_2_, and nuclear staining was performed with Mayers’ hematoxylin. The immunohistochemical staining of each individual antigen was blindly evaluated by pathologists using a semi-quantitative method: the percentage of stained cells and signal intensity score (0–3).

##### Antibody coding sequence isolation and analysis

The coding sequences from the antibodies produced by the selected hybridomas – AM51.1 and AM52.1 clones – were isolated as previously described.^63^ Briefly, the hybridomas were grown and when the culture reached 1 million cells, pellets were prepared and lysed in mRNA extraction buffer (Absolutely RNA Miniprep, Agilent Technologies, USA). Total mRNA was converted into cDNA by reverse transcription, and subsequently 5′-dC tailed and extracted by ethanol precipitation. The resulting product was then amplified in a serial nested PCR using GI oligo, a universal dC-annealing primer, and constant part-specific primers. The PCR reactions were subcloned into a T/A cloning system. Single bacterial colonies were picked and insert-containing vectors were sent for sequencing (*Eurofins Genomics,* GmbH, Ebersberg, *Germany*). The sequences of interest were compared against the public IMGT database (http://www.imgt.org/IMGT_vquest/vquest) to identify the variable chains, hypervariable domains, and somatic mutations.

#### Chimeric antigen receptor T cells

##### Vector design

The scFvs were built based on AM51.1 and AM52.1 starting with a secretion sequence (L chain) followed by the light chain variable fragment (V_L_) linked to the variable heavy chain by four glycine-serine repeats [(G_4_S)_4_]. The protein sequences were ordered as synthetic DNA (*Eurofins Genomics*), cloned into a second-generation signaling tail-containing vector (pENTR) composed of a CD8 hinge and transmembrane domains linked to a 4-1BB and CD3ζ. The tCD34 isoform was inserted downstream of the CAR construct separated by a P2A ribosome skipping sequence to enable convenient evaluation of the expression. Verified clones were recombined into compatible expression vectors using the Gateway cloning system (Thermo Fisher Scientific). FMC63 mAb-based CAR (CD19CAR) and humanized CC49 (huCC49)-based CAR (TAG72CAR) were designed in the same scaffold as for the AM antibodies-based CARs. CD19 scFv was a kind gift of Martin Pule (UCL Cancer Institute, London, UK), and TAG72 scFv was obtained from Kashmiri et al.^32^

##### In silico analysis of the single-chain variable fragments

The sequences of the scFvs built from the different antibodies were aligned using MAFFT^65^ with default parameters and visualized in Jalview.^66^ The antibody structures were computationally generated using ABodyBuilder2^33^ and IgFold^34^; however, ABodyBuiler2 structures were utilized for comparison and 3D representation among the antibodies. Using ChimeraX (version 1.6.1),^67^ the scFvs were then structurally aligned, guided by an initial sequence to ensure optimal 3D spatial alignment of corresponding regions. The sequence alignment was performed using the Needleman-Wunsch algorithm with BLOSUM-62 scoring, incorporating a 30% weighting for secondary structure elements. RMSD values were computed across all pairs without pruning. CDRs were identified using ANARCI^68^ with the IMGT numbering scheme.^69^

##### Transduction and expansion

HEK-293-Phoenix cells (1.5 × 10^6^) previously plated in 6 cm plates were transfected using XtremeGene9 (Roche) with a DNA mix including retroviral packaging vectors, the expression vector to an equimolar ratio, and a GFP reporter vector. After 24h, the medium was replaced with 1% HyClone FBS-containing DMEM and cells were transferred to a 32°C incubator. The retroviral supernatants were harvested 48 and 72 h post-transfection. PBMCs isolated from healthy donors were resuspended in X-VIVO 15 (Lonza Bioscience, Walkersville, MD, USA) supplemented with 100 U/mL recombinant human IL-2 (Proleukin, Clinigen Healthcare Ltd, UK) and 5% of human serum (HS; PAN-Biotech GmbH, Germany), and transferred to a 24-well plate coated with anti-CD3 (1 μg/mL; Thermo Fisher Scientific) and anti-CD28 (1 μg/mL; Thermo Fisher Scientific) at 1 × 10^6^ cells/well. Activated T cells were subsequently transduced with viral supernatant 3 days after activation using spinoculation of 750 × g for 60 min at 32°C in a 24-well non-treated plate (Nunc A/S) pre-coated with retronectin (50 μg/mL; Takara Bio, *Shiga*, *Japan*). Transduction efficiency was assessed after 3 to 7 days by flow cytometry using biotinylated goat anti-mouse IgG (fragment antigen binding (Fab) specific) antibody (dilution 1:200; Jackson Immunoresearch) followed by a secondary staining with streptavidin-PE (0.5 μg/mL; Biolegend, San Diego, CA, USA), and APC-conjugated antibody against human CD34 (1 μg/mL; Thermo Fisher Scientific). Transduced CAR T cells were counted and expanded at densities of 1 × 10^6^ cells/mL over a period of 10 days, or restimulated with CD3/CD28 Dynabeads (Thermo Fisher Scientific) at a 1 T cell: 1 bead ratio. At the end of the expansion, in case of restimulation (day 16), expanded T cells were counted again, frozen in batches and stored in liquid nitrogen for future experimental use.

##### Reporter assay

The J-76 cell line was transduced with a nuclear factor of activated T cells (NFAT)-green fluorescent protein (GFP) reporter construct,^70^ and a single clone was isolated (J-76-NFAT-GFP).^71^ J-76-NFAT-GFP were subsequently transduced with the CAR vectors and then co-cultured with the indicated target cells at an E:T ratio of 1:2 in complete RPMI 1640 (Gibco). Cells were incubated at 37°C for 24h and CAR-mediated activation was assessed by GFP expression, as measured by flow cytometry. Effector cells incubated without targets or effectors in the presence of phorbol myristate acetate (PMA)/ionomycin were used to detect baseline and maximal activation, respectively.

##### Cytotoxicity assay

Virally transduced GFP- and luciferase-expressing (GFP^+^/Luc^+^) target cells were counted, resuspended in phenol red-free RPMI 1640 (Biowest, Riverside, MO, USA) at a concentration of 3 × 10^5^ cells/mL, and seeded (100 μL/well) in triplicate in a white flat-bottomed 96-well plate (Thermo Fisher Scientific) in the presence of D-Luciferin (75 μg/mL; PerkinElmer, Waltham, MA, USA). After 24h, CAR T cells were added to the wells at the indicated E:T ratio. Target cells incubated without effectors or in the presence of 1% Triton X-100 (Sigma-Aldrich) were used to detect baseline lysis and maximal killing, respectively. Cells were incubated at 37°C and the bioluminescence was measured as relative light units (RLUs) at indicated time points with a VICTOR Multilabel Plate Reader (PerkinElmer). Lysis percentage was calculated using the following equation: % specific lysis = 100 × ((spontaneous cell death RLU - sample RLU)/(spontaneous death RLU - maximal killing RLU)).

##### Degranulation assay

STn-positive and -negative target cells were seeded (100 μL/well) in a 96-well plate (Thermo Fisher Scientific) at 5 × 10^4^ cells/well in plain X-VIVO 15 medium (Lonza Bioscience). After 24h, CAR T cells were added at an E:T ratio of 1:2 in the presence of anti-CD107a-PE-Cy5a mAb (6.3 μg/mL; BD Biosciences), GolgiPlug, and GolgiStop (dilution 1:1000; BD Biosciences). Co-cultures were incubated at 37°C for 6h. Following incubation, cells were stained for CD4 and CD8 (0.14 and 0.2 μg/mL, respectively; BD Biosciences), and T cell degranulation was assessed by measuring CD107a expression on CD4^+^ and CD8^+^ cells using flow cytometry. Effector cells cultured alone (baseline) and those stimulated with PMA/ionomycin (maximal activation) were included as negative and positive controls, respectively.

##### Cytokine release assay

CAR T cells were co-cultured for 24h with the indicated cell lines at an E:T ratio of 1:1 in plain X-VIVO 15 medium (Lonza Bioscience). Culture supernatants were harvested, centrifuged to remove cells and debris, and diluted 1:2 prior to cytokine analysis. Cytokine levels were measured using the Bio-PlexPro Human Cytokine 17-plex Assay (Bio-Rad Laboratories Inc.) according to manufacturer’s instructions. Effector-only and effector-only cultures were included to establish baseline cytokine levels, and co-cultures of CD19CAR T cells with CD19-positive BL-41 cells served as a positive control. Cytokines were quantified using the Bio-Plex 200 system (Bio-Rad Laboratories Inc.), and concentrations were determined from a standard curve using Bio-Plex Manager software version 6.1.

##### Patient-derived organoid studies

Gastric PDOs were extracted from Matrigel (Corning) using Cell Recovery Solution (Corning, New York, United States) at 4°C for 45 min. PDOs were washed with culture media and dissociated into single cells using TrypLE (GIBCO) at 37°C. Cells were counted and seeded into either a 96-well flat bottom plate (Thermo Fisher Scientific) for the viability assay or a black 96-well ibiTreat flat, square bottom plate (ibidi) pre-coated with 15% Matrigel for immunostaining. CAR T cells were subsequently added to the wells at the indicated E:T ratios and incubated at 37°C for 24h. For immunofluorescent detection, PDOs were fixed with 4% PFA (Thermo Fisher Scientific) for 30 min at RT, followed by permeabilization with NH4Cl 50 mM/PBS for 30 min at RT, and with 0.1% Triton X-100/PBS for 30 min at RT. Blocking was performed with 10% BSA/PBS for 30 min at RT, followed by ON incubation with the anti-CC3 antibody (dilution 1:200; Cell Signaling Technology, Danvers, MA, United States) at 4°C. PDOs were subsequently labeled with Alexa Fluor 488-conjugated secondary antibody (2 μg/mL; Thermo Fisher Scientific) for 45 min at RT, and underwent nuclear staining with Phenovue Hoechst 33342 (Novabio, Kaunas, Lithuania). Images were acquired with an Opera Phenix Plus (PerkinElmer). To evaluate organoid viability, the co-culture media was aspirated, and 50 μL of fresh plain medium and 100 μL of CellTiter-Glo 3D Reagent (VWR International) were added. Subsequently, PDOs were mechanically disrupted, and the mixture was incubated for 25 min at RT. Luminescence signal was quantified through a SynergyMxTM MultiMode Microplate Reader (BioTekTM). Viability values were normalized to the Mock-treated conditions, with each PDO considered to have 100% viability under those conditions. Treatment of PDOs with 20 mM MG132 (Selleckchem, Houston, United States) was used as a positive control of the assay.

##### *In vivo* experiments

Tumor xenografts were established in 6- to 8-week-old NXG mice by ip administration of 2.5, 1, or 10 × 10^6^ GFP/Luc^+^ OV-90, OVCAR-3, or SNU-16 cells, respectively, or with 250 μL of mucinous tumor tissue from a CRC patient with mucinous peritoneal metastases, as previously established.^36^^,^^62^ Tumor-bearing mice received 2 IP injections of 5 × 10^6^ CAR T cells or non-transduced T cells (Mock group) at the indicated time points. Tumor progression in mice bearing GFP/Luc-expressing human cell lines was monitored by bioluminescence imaging (BLI) with an *in vivo* imaging system (IVIS, Lumina III; PerkinElmer), and image analysis was performed using the Xenogen Spectrum system and Living Image 3.2 software (LI-COR Biosciences, Nebraska, USA). For mice engrafted with patient-derived tumor tissue, tumor burden was monitored by magnetic resonance imaging (MRI).

### Quantification and statistical analysis

Statistical analyses were performed using GraphPad Prism v9.0. Data are displayed as mean ± standard deviation (SD). Statistical comparisons were performed using Student’s t test or two-way ANOVA, as appropriate. For *in vivo* studies, survival curves were compared using the log rank (Mantel-Cox) test. Unless otherwise specified, all comparisons were made relative to the Mock control group. In all tests, *p* values ≤0.05 were considered statistically significant (^∗^ *p* ≤ 0.05; ^∗∗^ *p* ≤ 0.01; ^∗∗∗^ *p* ≤ 0.001; ^∗∗∗∗^ *p* ≤ 0.0001), with 95% confidence interval. The exact sample size (*n*) and description (e.g., number of donors) are indicated in each figure legend. Additional details on statistical tests and replicates are provided in the relevant figure legends.

## Supplemental information

Document S1. Figures S1–S6 and Tables S1 and S2

Document S2. Article plus supplemental information
