# Supplementary material for: Pan-carcinoma sialyl-Tn-targeting expands CAR therapy to solid tumors
Source: Cell Rep Med. 2025 Sep 8;6(9):102350. doi: 10.1016/j.xcrm.2025.102350 (PMC12490251; doi:10.1016/j.xcrm.2025.102350)
Supplement: Document S1. Figures S1–S6 and Tables S1 and S2 [file mmc1.pdf]

Cell Reports Medicine, Volume *6*

Supplemental information

Pan-carcinoma sialyl-Tn-targeting expands CAR therapy to solid tumors

Rafaela Abrantes, Christopher Forcados, David J. Warren, Liliana Santos-Ferreira, Karianne Giller Fleten, Emanuel Senra, Ana Filipa Costa, Klara Krpina, Rui Henrique, Ann Magritt Liberg, Puneet Rawat, Pascal Gelebart, Emmet McCormack, Line Bjørge, Ben Davidson, Victor Greiff, Daniela Elena Costea, Filipe Pinto, Kjersti Flatmark, Catarina Gomes, Else Marit Inderberg, Celso A. Reis, and Sébastien Wälchli

Pan-Carcinoma Sialyl-Tn-Targeting Expands CAR Therapy to Solid Tumors

Supplementary Material

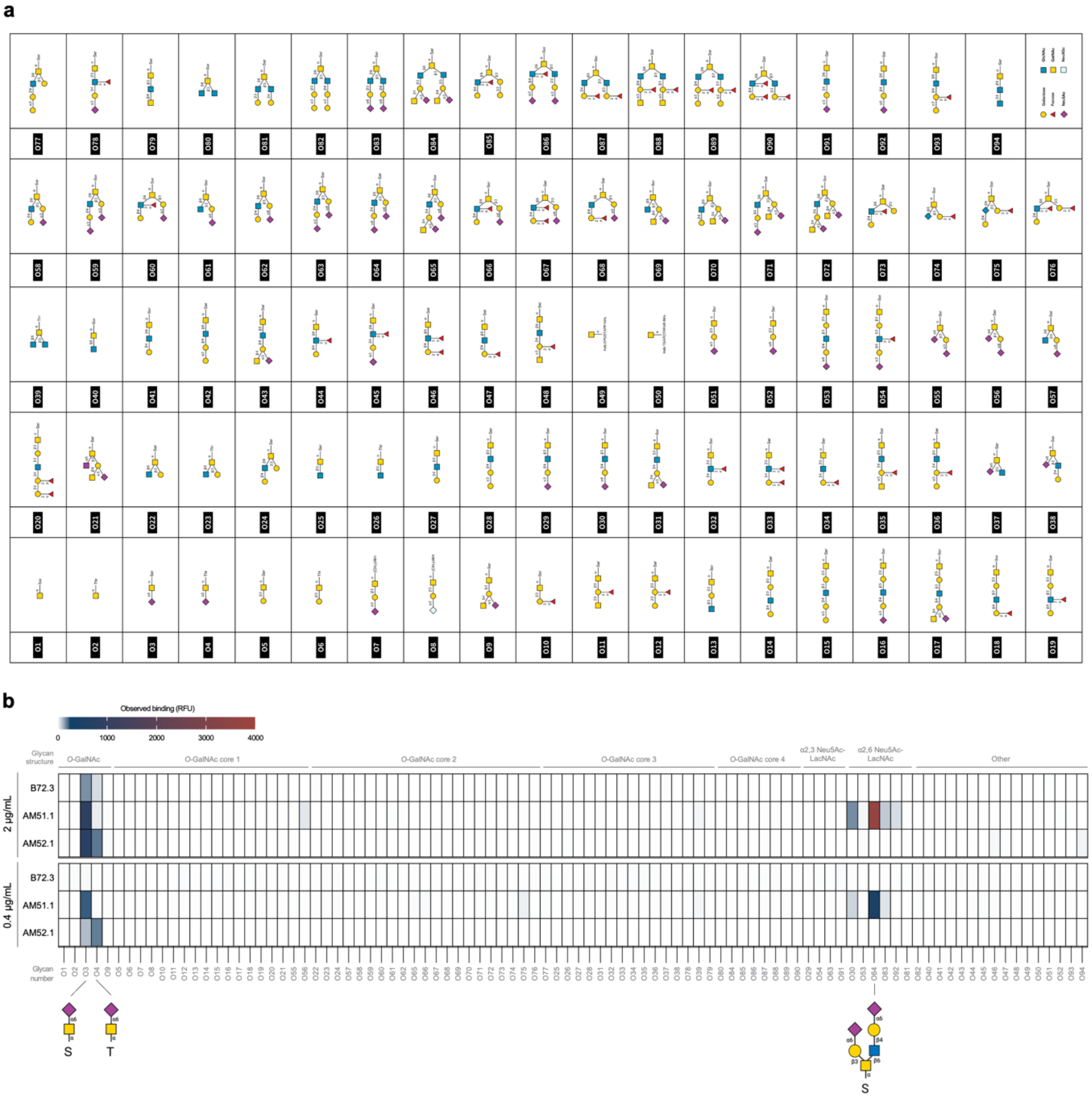

Supplementary Figure 1. Glycan microarray analysis of the novel AM51.1 and AM52.1 mAbs. a, List of the 94 *O*-glycan structures related to Sialyl-Tn (STn) tested in the glycan microarray. **b,** Two different concentrations (2 and 0.4 µg/mL) of the AM antibodies were tested against various *O*-glycan structures related to STn. B72.3 was included as assay control. Related to Figure 1.

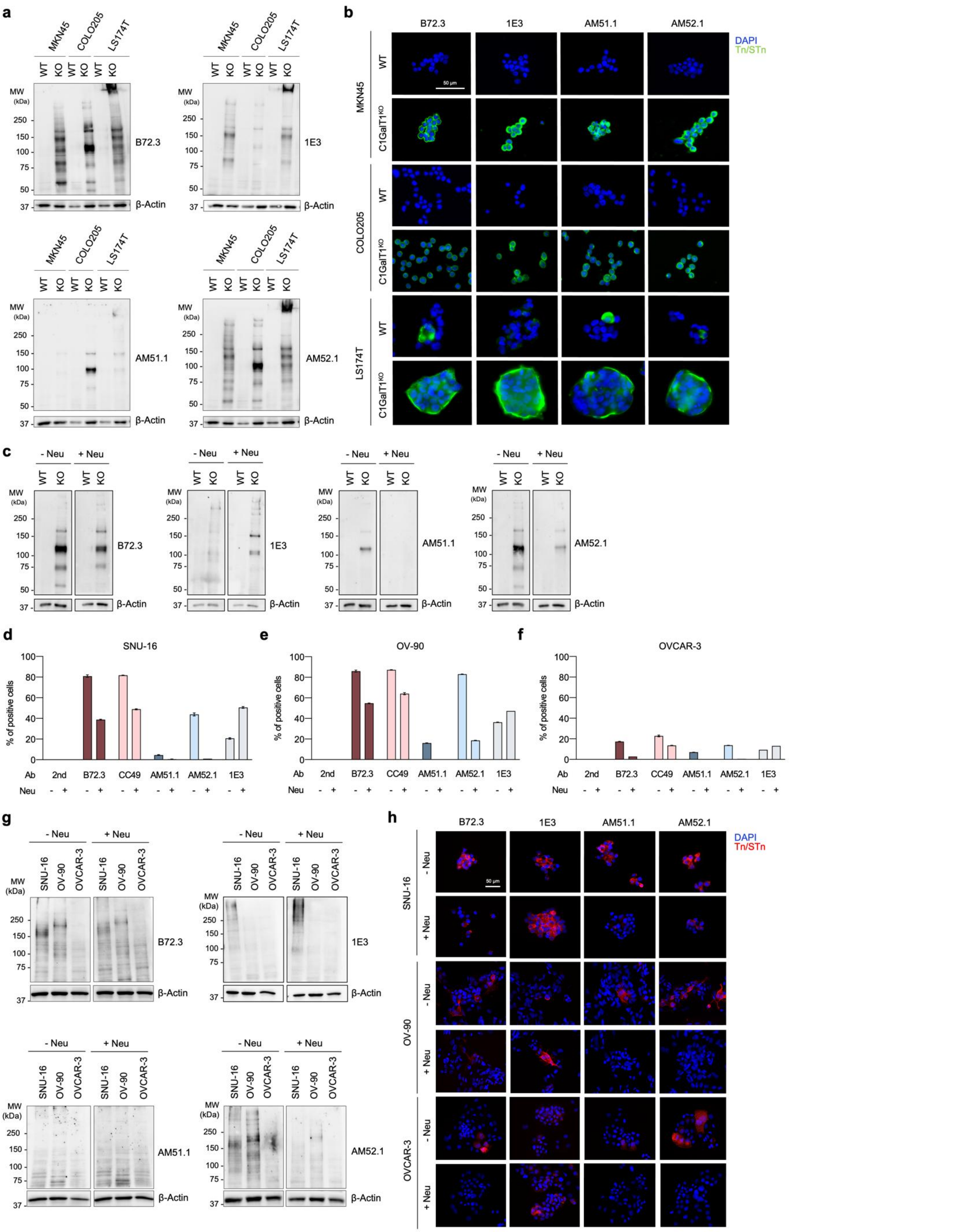

Supplementary Figure 2. AM51.1 and AM52.1 specifically bind to Sialyl-Tn in glycoengineered and naturally expressing cancer cells. a, Western blotting analysis and **b,** immunofluorescent labeling of gastrointestinal cancer wild-type (WT) and core 1 β1,3-galactosyltransferase (C1GalT1) knock-out (KO) cells by the AM mAbs. B72.3 and 1E3 were used as controls. **c.** Confirmation of the AM mAbs binding specificity by Western blotting analysis against WT and C1GalT1^{KO} COLO205 cell lysates, with or without pretreatment with neuraminidase. **d,** Gastric and **e and f,** ovarian cancer cell lines endogenously expressing Sialyl-Tn (STn) were stained for flow cytometry, with and without prior neuraminidase treatment, using the AM antibodies, along with antibody controls. **g,** Western blotting analysis and **h,** immunofluorescent labeling of SNU-16, OV-90, and OVCAR-3 cells by the AM mAbs, with or without pretreatment with neuraminidase. B72.3 and 1E3 were used as controls. Scale bars correspond to 50 μm. Related to Figure 1.

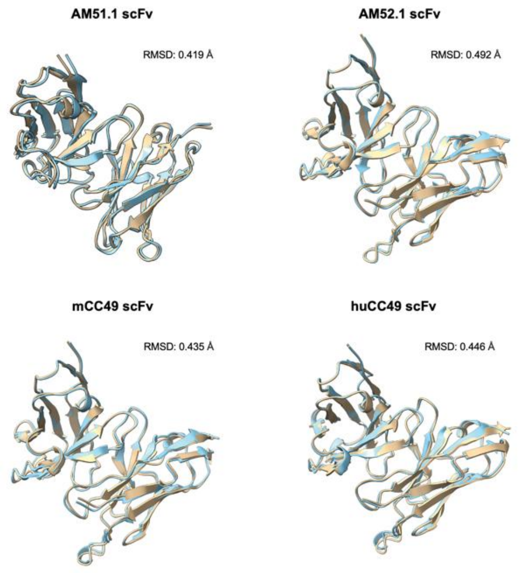

Supplementary Figure 3. The *in silico* structures of the single-chain variable fragment domains demonstrate consistent alignment across different computational methods. Structures were generated using ABodyBuilder2 (beige color) and IgFold (cyan color), and subsequently aligned using ChimeraX (version 1.6.1). The corresponding root mean square deviation (RMSD) values are provided alongside each structure. Related to Figure 2.

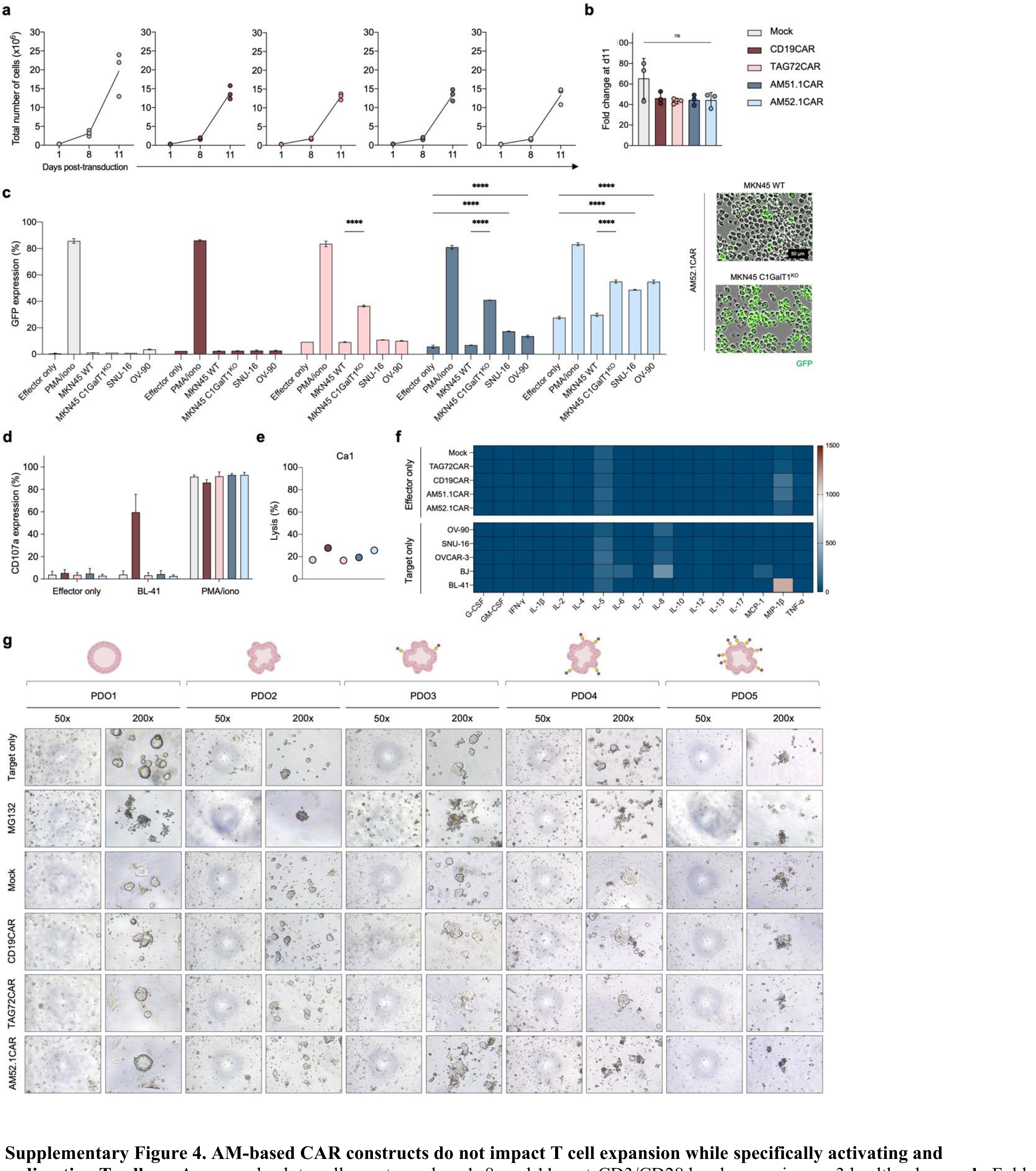

Supplementary Figure 4. AM-based CAR constructs do not impact T cell expansion while specifically activating and redirecting T cells. a, Average absolute cell counts on days 1, 8, and 11 post-CD3/CD28 bead expansion. n=3 healthy donors. **b,** Fold change in T cell counts, calculated as the cell count on day 11 (d11) divided by the cell count on day 1. n=3 healthy donors. **c,** J-76-NFAT-GFP cells transduced with different CAR constructs were co-cultured with cancer cells expressing various Sialyl-Tn (STn) levels. GFP expression, serving as an indirect measure of T cell activation, was quantified by flow cytometry. Representative fluorescence images show co-cultures of AM52.1CAR T cells with MKN45 wild-type (WT) or core 1 β1,3-galactosyltransferase (C1GalT1) knock-out (KO) cells. Scale bar corresponds to 50 µm. **d,** Degranulation activity of $CD4^+$ and $CD8^+$ after 24h co-culture alone (effector only), with STn-negative target cells (BL-41), or stimulated with phorbol myristate acetate (PMA) and ionomycin (maximal activation), at an Effector:Target (E:T) ratio of 1:2. n=3 healthy donors. **e,** Flow cytometry-based killing assay of CAR T cells co-cultured with the STn-negative cancer cell line Ca1 at an Effector:Target (E:T) ratio of 10:1 for 24h. Data correspond to the 24-hour time point. n=1 healthy donors. **f,** Cytokine secretion following 24-hour culture of effector or target cells alone to establish baseline cytokine levels. n=3 healthy donors. **g,** Morphological analysis of patient-derived organoids (PDOs) observed under a light microscope after 24h of co-culture with the indicated CAR T cells at an Effector:Target (E:T) ratio of 10:1. Representative images are shown at two magnifications: 50x and 200x. Data are represented as mean +/- standard deviation (SD). Statistical significance was determined using paired *t*-test. * $p \leq 0.05$; ** $p \leq 0.01$; *** $p \leq 0.001$; **** $p \leq 0.0001$. Related to Figure 3.

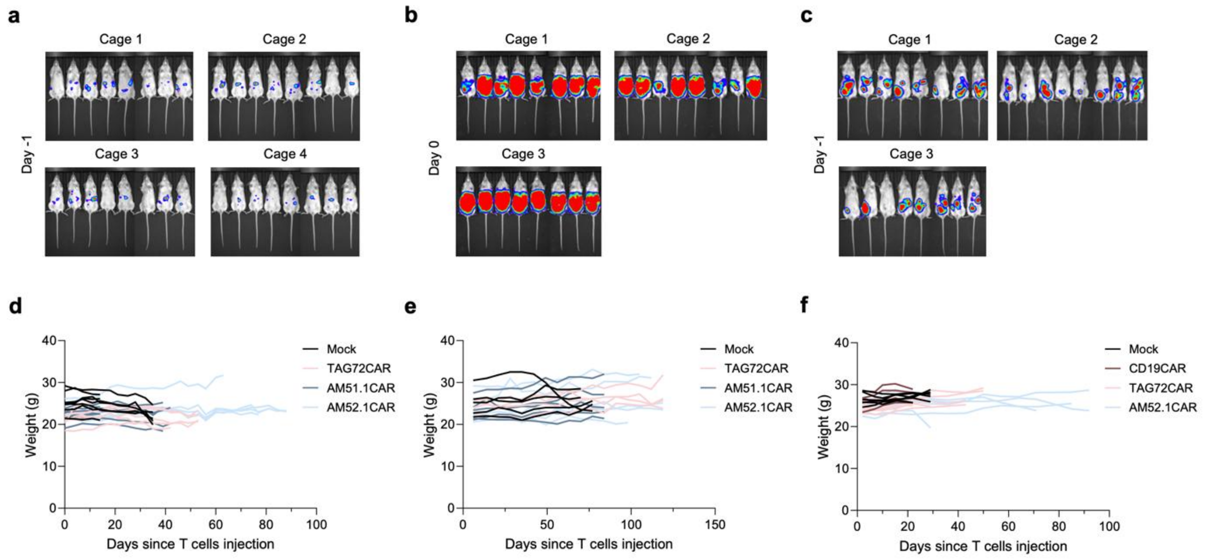

Supplementary Figure 5. Randomization-day images and body weight monitoring of xenograft mouse models. Representative images of mice after randomization for the **a,** OV-90; **b,** OVCAR-3; and **c,** SNU-16 xenograft models. Body weight monitoring during the *in vivo* experiments for the **d,** OV-90; **e,** OVCAR-3; and **f,** SNU-16 xenograft models. Related to Figure 5.

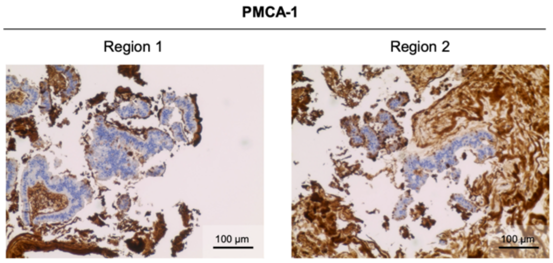

Supplementary Figure 6. AM52.1 staining of mucinous peritoneal metastasis tissue from the colorectal cancer patient selected for the establishment of the patient-derived xenograft model. Immunohistochemical staining of two distinct representative regions of the peritoneal mucinous carcinomatosis from patient 1 (PMCA-1) are shown. Scale bars correspond to 100 µm. Related to Figure 6.

Supplementary Table 1. Immunohistochemical evaluation of various carcinomas using the AM monoclonal antibodies. For each antibody and lesion, results are shown as the percentage of tumor cells staining positively (%) and the corresponding staining intensity (scored from 1 (weak) to 3 (strong)). Each row represents an individual patient sample (n). Related to Figure 1.

		ANTIBODIES					
		B72.3		AM51.1		AM52.1	
LESION	n	Percentage	Intensity	Percentage	Intensity	Percentage	Intensity
Gastric cancer	1	60-70	3	60-70	3	20-30	1
	2	30-40	3	0	0	0	0
	3	80-90	3	10-20	2	80-90	3
	4	10-20	3	0	0	10-20	3
Colorectal cancer	1	50-60	2	10-20	2	1-10	2
	2	70-80	3	70-80	3	70-80	2
	3	90-100	3	90-100	3	70-80	2
	4	1-10	1	0	0	0	0
	5	60-70	2	0	0	0	0
Pancreatic cancer	1	90-100	3	20-30	3	60-70	3
	2	70-80	1	<1	1	<1	2
	3	1-10	3	1-10	2	1-10	3
	4	10-20	2	1-10	1	30-40	3
	5	20-30	1	20-30	1	<1	1
Prostatic cancer	1	0	0	0	0	0	0
	2	20-30	3	0	0	10-20	3
	3	<1	1	0	0	0	0
	4	1-10	2	0	0	10	2
	5	1-10	2	0	0	10	1
Ovarian cancer	1	10-20	1	<1	1	10-20	1
	2	30-40	2	1-10	1	30-40	2
	3	30-40	2	<1	1	20-30	2
	4	<1	1	0	0	<1	1
	5	1-10	1	0	0	1-10	1

Supplementary Table 2. Prediction errors obtained for each region of the single-chain variable fragment from ABodyBuilder2. The prediction error for each region was calculated by taking the RMS of the individual prediction errors for each residue within that region. Related to Figure 2.

	ANTIBODIES			
**REGION**	**AM51.1**	**AM52.1**	**mCC49[e]**	**huCC49[c]**
Framework (H-chain[b])	0.25	0.32	0.27	0.30
CDR[a]-H1	0.32	0.28	0.29	0.20
CDR-H2	0.18	0.16	0.24	0.22
CDR-H3	0.20	0.18	0.18	0.16
Framework (L-chain[d])	0.23	0.18	0.22	0.18
CDR-L1	0.24	0.37	0.25	0.30
CDR-L2	0.18	0.15	0.26	0.22
CDR-L31	0.24	0.17	0.21	0.23

Abbreviations: [a]Complementarity-determining region; [b]Heavy-chain; [c]Humanized CC49; [d]Light-chain; [e]Murine CC49
